# Comparative organelle genome analysis of three medicinal species of the genus *Woodwardia* (Blechnaceae): new insights into the phylogeny and molecular identification

**DOI:** 10.3389/fpls.2026.1905066

**Published:** 2026-10-07

**Authors:** Yutong Huang, Libao Feng, Yanping Xing, Wenxiao Men, Yanchang Huang, Tingguo Kang, Yanyun Yang, Liang Xu

**Affiliations:** 1 School of Pharmacy, Liaoning University of Traditional Chinese Medicine, Dalian, China; 2 Traditional Chinese Medicine Quality and Resource Development Professional Technology Innovation Center, Liaoning Provincial Department of Science and Technology, Shenyang, China

**Keywords:** chloroplast genome, mitochondrial genome, molecular identification, phylogeny, *Woodwardia*

## Abstract

**Introduction:**

Plants of the genus *Woodwardia* have a long history of medicinal use in China. Among these plants, *Woodwardia japonica, Woodwardia unigemmata*, and *Woodwardia prolifera* are the primary source species of Guan Zhong, a traditional Chinese medicine. However, because of morphological similarity among their dried herbal materials and limited availability of organellar (mitochondrial and chloroplast) genomic data, research on species identification, molecular evolution, and phylogeny has been impeded.

**Methods:**

Organelle genomes of the three *Woodwardia* species were sequenced and assembled using the second- and third-generation sequencing technologies. In-depth analyses were conducted, including protein-coding genes (PCGs) presence/absence, non-synonymous/synonymous mutation ratios (Ka/Ks), codon usage bias, repeat sequence identification, RNA editing site prediction, synteny and mitochondrial–chloroplast homologous fragment (MTPT) identification, and molecular identification via mVISTA and nucleotide diversity. Finally, we employed both maximum likelihood (ML) and Bayesian (BI) methods, based on single-copy orthologous genes and secondary calibrations, to infer phylogenetic relationships and divergence times among *Woodwardia* and related ferns.

**Results:**

The mitochondrial genomes of the three *Woodwardia* species exhibited notable differences in genome size and characteristics; while the chloroplast genomes were relatively conserved and highly similar. Phylogenetic and divergence time analyses indicated that *W. unigemmata* and *W. prolifera* are more closely related and diverged more recently. Integrated analyses of highly variable regions and phylogenetic revealed that the intergenic spacer *rrn16–rps12_exon2 or rps12_exon2–rrn16* could effectively differentiate the three Guan Zhong-related *Woodwardia* species, suggesting their potential utility as reliable DNA barcodes for identifying medicinal materials.

**Discussion:**

The results provide comprehensive insights and reliable, detailed organelle genomic data for the phylogeny and molecular identification of W. japonica, *W. unigemmata*, and *W. prolifera*.

## 1 Introduction

The genus *Woodwardia* (family Blechnaceae) comprises fern species distributed from temperate to subtropical regions of Asia, Europe, and the Americas. To date, approximately 12 species of *Woodwardia* have been identified worldwide, 5 of which occur in China, including *Woodwardia japonica* (L. f.) Sm., *Woodwardia unigemmata* (Makino) Nakai, and *Woodwardia prolifera* Hook. & Arn (Flora of China Editorial Committee, 2013). *Woodwardia* plants have a long history of medicinal use in China. Notably, *W. japonica*, *W. unigemmata*, and *W. prolifera* serve as the primary plant sources for preparing the traditional Chinese medicine Guan Zhong in several provinces of southern China. Traditionally, these plants have been utilized for heat removal, detoxification, and parasite expulsion and are commonly used to treat influenza, dysentery, and related disorders (Editorial Committee of Zhonghua Bencao, 1999). Modern pharmacological studies indicate that *Woodwardia* species are rich in flavonoids and triterpenoids and exhibit remarkable antibacterial and antiviral activities, among other bioactive properties (Ma et al., 2017; Wang et al., 2023; Takuli et al., 2020).

From a taxonomic perspective, *Woodwardia* species display considerable variations in plant size and leaf texture due to differences in habitat conditions. In recent decades, several new species and varieties have been proposed within the genus *Woodwardia*; however, many of these reports were found to be invalid because of reliance mostly on morphological traits observed in robust, large-sized individuals (Boonkerd and Pollawatn, 2004; Chiu, 1974). Thus, integrative systematic studies combining detailed morphological analyses with genomic approaches offer an effective strategy to prevent such misidentifications in the future. In the context of medicinal material identification, *W. japonica*, *W. unigemmata*, and *W. prolifera* are the source species of Guan Zhong. Their dried herbal slices exhibit highly similar macroscopic features (**Supplementary Figure S1**), and currently, their differentiation relies primarily on morphological characteristics. Although previous studies have applied universal DNA barcodes (e.g., *psbA–trnH*) for the molecular identification of Guan Zhong-type medicinal materials and achieved satisfactory outcomes (Cai et al., 2016), no species-specific DNA barcode has yet been developed for these three *Woodwardia* medicinal species. Therefore, identifying exclusive DNA barcodes that can accurately differentiate these three *Woodwardia-*derived medicinal materials represents a crucial direction for enhancing the accuracy and reliability of their authentication.

Mitochondria and chloroplasts in plant cells are semi-autonomous organelles. They possess genetic systems independent of the cell nucleus, and the sequences of their functional genes are highly conserved in evolution (Palmer and Herbon, 1988). Mitochondrial genomes are prominently characterized by their high interspecific diversity, including substantial variation in genome size, structure, and other features (Mower et al., 2012). In contrast, chloroplast genomes are highly conserved in terms of sequence structure and are relatively stable in size (Palmer, 1985). Thus, this feature of strong conservation and the distinct evolutionary rates compared to those of nuclear genes make mitochondrial and chloroplast genomes valuable tools for molecular evolutionary and ecological studies (Zhang et al., 2021). *W. japonica*, *W. unigemmata*, and *W. prolifera* are typical representatives of *Woodwardia* species in terms of natural reproductive strategies, and they collectively encompass all known reproductive modes within the genus, including reproduction through spores, gemmae, and bulbils (**Figure 1A, B, C**). Comparative analyses of organelle genomes among congeneric species provide multiple perspectives for understanding plant evolution and facilitate the development of genus-specific DNA barcodes essential for the accurate identification of *Woodwardia* species.

**Figure 1:**
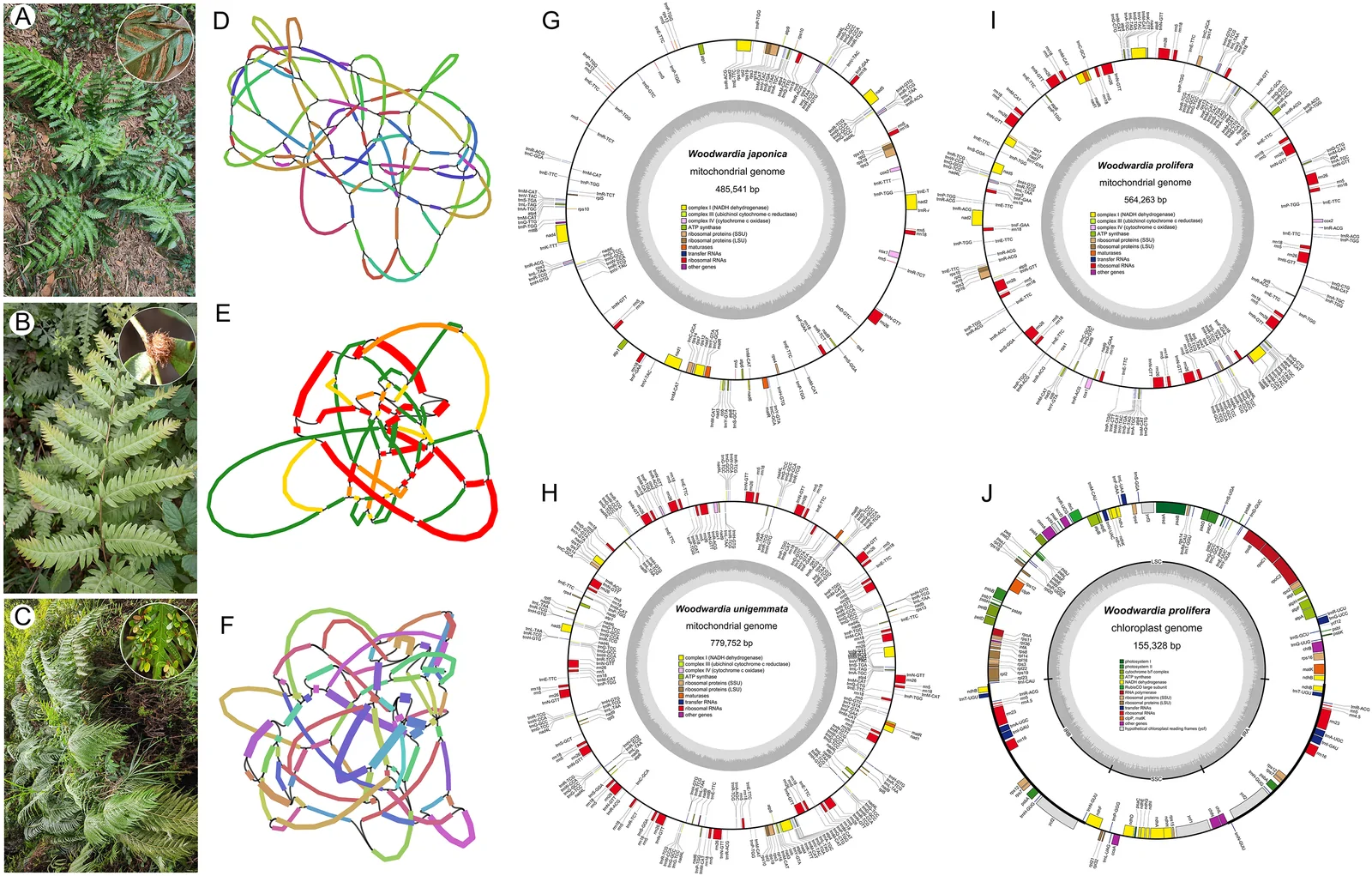
Schematic diagram and gene map of mitochondrial genomes of three *Woodwardia* species and the chloroplast genome assembly of *W. prolifera.* **(A, D, G)** *W. japonica;* **(B, E, I)** *Woodwardia orientalis;* **(C, F, H, I)** *W. prolifera.* **(A–C)** Photographs of the original plant materials. **(D–F)** GFA graphs supported by PacBio Revio HIFI data, visualized using Bandage software. **(C, F, H, I)** Genes located inside the circle represent transcription in the clockwise direction, while those outside the circle represent the opposite direction. Genes with different functions are marked in different colors. The inner gray histogram shows the GC content of the genome, and the central gray line indicates the 50% threshold.

There have been no reports of fully assembled nuclear genomes for species of *Woodwardia*. Taking the NCBI database as an example, only a small number of partial nuclear gene sequences (e.g., ITS2) from *W. japonica* and *W. unigemmata* are currently available. Most published studies on *Woodwardia* have focused on chloroplast genomes; nevertheless, complete chloroplast genome assemblies have only been reported for *W. japonica* and *W. unigemmata* (Wei et al., 2017; Ramekar et al., 2020). When Ramekar et al. reconstructed the evolutionary relationships of *W. japonica* using chloroplast genome data, only *W. unigemmata* from the genus *Woodwardia* was included in the phylogenetic tree. Overall, substantial gaps remain in the sequencing, assembly and phylogenetic analyses of organellar genomes in *Woodwardia.* In particular, no mitochondrial genomes of this genus have been sequenced or publicly released to date. Therefore, the present study aimed to use second- and third-generation sequencing technologies to perform, for the first time, sequencing, assembly, and comparative analyses of the mitochondrial genomes of *W. japonica*, *W. unigemmata*, and *W. prolifera* and the chloroplast genome of *W. prolifera*. Specifically, we sought to (1) compare the structural features and genomic characteristics of mitochondrial and chloroplast genomes among the three *Woodwardia* species; (2) explore genomic divergence and patterns of adaptive evolution in the mitochondrial and chloroplast genomes of these three species; (3) infer phylogenetic relationships among the three *Woodwardia* species; (4) estimate the divergence times of the three species based on complete chloroplast genome sequences; and (5) identify highly variable regions suitable as potential DNA barcodes for the reliable molecular identification of these species. By conducting these analyses, we attempted to elucidate the phylogenetic relationships among *W. japonica*, *W. unigemmata*, and *W. prolifera* from the perspective of organelle genomes and provide comprehensive genetic resources to support the accurate identification, sustainable development, and utilization of these three *Woodwardia*-derived medicinal materials.

## 2 Materials and methods

### 2.1 Plant materials and genome sequencing

Fresh, healthy, and young green leaves were collected as experimental materials from Leshan City, Sichuan Province, and Heyuan City, Guangdong Province, China. The species were identified by Prof. Liang Xu (Liaoning University of Traditional Chinese Medicine) as *W. japonica* (103°25′18.92″E, 29°31′52.04″N), *W.unigemmata* (103°25′18.85″E, 29°31′47.40″N), and *W. prolifera* (115°24′13.55″E, 23°18′1.02″N).

Mitochondrial genomes: High-quality total DNA was extracted from fresh leaves by using a modified cetyltrimethylammonium bromide (CTAB) method (Doyle and Doyle, 1987). DNA purity and concentration were confirmed using a NanoDrop 1000 spectrophotometer and a Qubit fluorometer (Thermo Fisher Scientific, Shanghai, China), respectively. A 15-kb SMRTbell library was then constructed using the SMRTbell Express Template Prep Kit 2.0 (Pacific Biosciences, CA, USA). The library construction steps included DNA shearing, purification with AMPure PB magnetic beads, removal of single-stranded overhangs, damage repair, end repair, ligation with hairpin adapters, and final library purification using magnetic beads. After quality control assessment, a qualified SMRTbell library was obtained and sequenced on the PacBio Revio platform (Pacific Biosciences, CA, USA). Raw reads were processed using the CCS algorithm (version 6.0.0; parameters: minPasses, 3; minPredictedAccuracy, 0.99; maxLength, 21,000) to generate high-accuracy HiFi reads (Mitochondrial genome sequencing coverage maps of three *Woodwardia* species see **Supplementary Figures S2**–**S7**).

Chloroplast genome: High-quality total DNA was also extracted from fresh leaves by using the modified CTAB method. A paired-end sequencing library with an insert size of 350 bp was constructed using the Nextera XT DNA Library Preparation Kit (Illumina, San Diego, CA, USA). This library was then subjected to paired-end sequencing on the Illumina NovaSeq 6000 platform to generate raw chloroplast genome data.

### 2.2 Genome assembly and annotation

Mitochondrial genomes: PacBio HiFi reads (total data volume > 5 Gb, average read length > 15 kb) were assembled using PMAT (v1.5.3, parameter: -g 5G) (Bi et al., 2024). The resulting assembly graphs were visualized and manually curated using Bandage (v0.8.1) to obtain preliminary circular genome structures (Wick et al., 2015). Subsequently, HiFi reads were mapped back to the assembled sequences using minimap2 (Li, 2018), and the assemblies were polished with NextPolish (v1.3.1, https://github.com/Nextomics/NextPolish), followed by manual refinement to generate the final mitochondrial genome assemblies and to adjust the corresponding graph files. The complete mitochondrial genomes were annotated using MITOFY and MFANNOT (Alverson et al., 2010). Following annotation, submission-ready files were generated for deposition in GenBank (https://www.ncbi.nlm.nih.gov/genbank/), and mitochondrial genome maps were plotted using OGDRAW (v1.3.1, https://chlorobox.mpimp-golm.mpg.de/OGDraw.html). The assembled complete mitochondrial genome sequences of *W. japonica*, *W. unigemmata*, and *W. prolifera* have been deposited in GenBank under accession numbers PV999925, PV999926, and PV999927, respectively.

Chloroplast genome: Raw Illumina reads were quality-filtered and processed using the NGS QC Toolkit (v2.3.3) (Patel and Jain, 2012). High-quality reads were then assembled *de novo* into the chloroplast genome using SPAdes (v3.14.1, https://gensoft.pasteur.fr/docs/SPAdes/3.14.1/manual.html) (Bankevich et al., 2012). Genome annotation was performed using the PGA pipeline (Qu et al., 2019), with the chloroplast genome of *W. unigemmata* (NC028543) serving as the reference for *W. prolifera*. After annotation, submission files were generated for GenBank, and the annotated chloroplast genome map was drawn using OGDRAW (Greiner et al., 2019). The assembled complete chloroplast genome sequence of *W. prolifera* has been deposited in NCBI GenBank under accession number PQ300107.

### 2.3 Analysis of the presence and absence of protein-coding genes

Core gene distribution maps were constructed based on annotated gene inventories across species by using R v3.6.0.

### 2.4 Analysis of selective evolutionary pressure on PCGs (Ka/Ks analysis)

Pairwise comparisons were conducted among *W. japonica*, *W. unigemmata*, and *W. prolifera*. For each pair of species, homologous gene pairs were identified from the set of shared PCGs. In total, 31 mitochondrial genes were shared (*atp4*, *atp6*, *atp8*, *atp9*, *cob*, *cox1*, *cox2*, *cox3*, *matR*, *mttB*, *nad1*, *nad2*, *nad3*, *nad4*, *nad4L*, *nad5*, *nad6*, *nad7*, *nad9*, *rpl16*, *rpl2*, *rpl5*, *rps1*, *rps10*, *rps12*, *rps13*, *rps14*, *rps19*, *rps3*, *rps4*, *rps7*), and 84 chloroplast genes were shared (*accD*, *atpA*, *atpB*, *atpE*, *atpF*, *atpH*, *atpI*, *ccsA*, *cemA*, *chlB*, *chlL*, *chlN*, *clpP*, *infA*, *matK*, *ndhA*, *ndhB*, *ndhC*, *ndhD*, *ndhE*, *ndhF*, *ndhG*, *ndhH*, *ndhI*, *ndhJ*, *ndhK*, *petA*, *petB*, *petD*, *petG*, *petL*, *petN*, *psaA*, *psaB*, *psaC*, *psaI*, *psaJ*, *psbA*, *psbB*, *psbC*, *psbD*, *psbE*, *psbF*, *psbH*, *psbI*, *psbJ*, *psbK*, *psbL*, *psbM*, *psbN*, *psbT*, *psbZ*, *rbcL*, *rpl14*, *rpl16*, *rpl2*, *rpl20*, *rpl21*, *rpl22*, *rpl23*, *rpl32*, *rpl33*, *rpl36*, *rpoA*, *rpoB*, *rpoC1*, *rpoC2*, *rps11*, *rps12*, *rps14*, *rps15*, *rps16*, *rps18*, *rps19*, *rps2*, *rps3*, *rps4*, *rps7*, *rps8*, *ycf1*, *ycf12*, *ycf2*, *ycf3*, *ycf4*).

For homologous genes exhibiting sequence divergence, the longer sequence was retained for further analysis. Sequence alignment and processing were then aligned and formatted using ParaAT 2.0 with default parameters (Zhang et al., 2012). To evaluate lineage-specific selective pressure, we performed codon-based selection analyses using PAML v4.9 (Yang, 2007). Homologous gene sequences were aligned with MAFFT v7.313 under codon alignment mode, and ambiguous alignment regions were trimmed using Gblocks v0.91b (Katoh and Standley, 2013; Talavera and Castresana, 2007). The maximum-likelihood tree was constructed using IQ-TREE v3.0.1 and served as the topological constraint for codeml analyses (Nguyen et al., 2015). Two nested branch models were applied: the one-ratio model (M0), which assumes a single uniform ω (non-synonymous substitution rate (Ka)/synonymous substitution rate (Ks)) across all branches, and the two-ratio branch model, which assigns distinct ω values to the predefined foreground branch and background branches. Likelihood ratio tests (LRT) were used to statistically compare model fitness and identify significantly altered selective pressures on the target lineage. The Ka and Ks were calculated for each gene pair using KaKs_Calculator (v2.0, https://sourceforge.net/projects/kakscalculator2/) by using the YN method (Wang et al., 2010). Finally, the Ka/Ks value was computed and summarized for each gene pair.

### 2.5 Codon usage analysis

Coding sequences of the 31 shared mitochondrial genes and 84 shared chloroplast genes from the three medicinal *Woodwardia* species were extracted using Perl scripts. Codon usage bias in mitochondrial protein-coding genes was analyzed using CodonW (v1.4.4, http://codonw.sourceforge.net), and relative synonymous codon usage (RSCU) values were calculated. Plots were generated to assess biases in synonymous codon preferences. The results were visualized using R v3.6.0.

### 2.6 Repeat sequence analysis

Simple sequence repeats (SSRs) were identified using MISA (v1.0) with the following parameters: 1-10 2-5 3-4 4-3 5-3 6-3 (Thiel et al., 2003). Interspersed repeats were detected using REPuter (https://bibiserv.cebitec.uni-bielefeld.de/reputer/), with the minimum repeat size set to 30 bp.

### 2.7 Prediction of RNA editing sites

RNA editing events in mitochondrial protein-coding genes were predicted using PmtREP. RNA editing sites in chloroplast protein-coding genes were identified using the online tool PREPACT3 (http://www.prepact.de/prepact-main.php), with *Adiantum capillus-veneris* (AY178864) selected as the reference sequence and an E-value cutoff of 0.8; all other parameters were retained at their default settings.

### 2.8 Synteny analysis

Homologous sequences between mitochondrial genomes and chloroplast genomes were identified using BLASTn v2.9.0+ with the following parameters: evalue, 1e-5; word_size, 7 (Camacho et al., 2009) Homologous fragments with lengths ≥ 1000 bp were defined as conserved syntenic blocks and used to generate multiple synteny plots. The results were visualized using TBtools v2.119 (Chen et al., 2023a).

### 2.9 Identification of mitochondrial plastid DNA sequence

Homologous sequences between mitochondrial genomes and chloroplast genomes were identified using BLASTn v2.9.0+ with the following parameters: evalue, 1e-5; word_size, 7 (Camacho et al., 2009). Homologous fragments with lengths ≥ 1000 bp were retained as homologous sequence blocks and used to generate homology plots. The results were visualized using TBtools v2.119 (Chen et al., 2023a). This BLASTn-based approach identifies regions of high sequence similarity, but does not assess conserved gene order or gene adjacency, which are required for strict synteny.

### 2.10 Phylogenetic analysis

For phylogenetic analysis, single-copy orthologous genes were identified using OrthoFinder v2.3.14 (Emms and Kelly, 2019). In total, 11 single-copy orthologous genes were shared among mitochondrial genomes (*atp9*, *cob*, *cox1*, *cox2*, *nad1*, *nad2*, *nad3*, *nad4*, *nad4L*, *nad5*, *nad6*), and 40 single-copy orthologous genes were shared among chloroplast genomes (*atpB*, *atpE*, *atpF*, *atpI*, *ccsA*, *matK*, *ndhC*, *ndhD*, *ndhE*, *ndhI*, *ndhJ*, *ndhK*, *petB*, *petG*, *petL*, *psaA*, *psaC*, *psaI*, *psaJ*, *psbB*, *psbD*, *psbF*, *psbH*, *psbI*, *psbJ*, *psbK*, *psbN*, *psbT*, *psbZ*, *rbcL*, *rpl14*, *rpl16*, *rpl20*, *rpl32*, *rpl33*, *rpoC2*, *rps3*, *rps8*, *ycf12*, *ycf4*).

These single-copy orthologous genes were aligned individually using MAFFT v7.429 (Katoh and Standley, 2013). Regions with ambiguous alignments, particularly those containing gaps, were trimmed using Gblocks 0.91b (Castresana, 2000.). The filtered alignments were then concatenated to construct a phylogenetic tree. *Andreaea regularis* (NC068856) was designated as the outgroup for mitochondrial phylogeny, and *Alsophila spinulosa* (NC012818) served as the outgroup for chloroplast phylogeny.

Maximum-likelihood (ML) trees were inferred using IQ-TREE v1.6.1 (Nguyen et al., 2015). The best-fitting nucleotide substitution models were selected using ModelFinder implemented in IQ-TREE based on the Bayesian information criterion (BIC). The optimal models were SYM+G4 and GTR+F+R4 for the mitochondrial and chloroplast datasets, respectively. The number of bootstrap replicates was set to 1000.

### 2.11 Divergence time estimation

Species divergence times are typically estimated using fossil calibrations or previously reported divergence times of related taxa, which serve as reference points for inferring the evolutionary divergence times of the species under investigation. In this study, divergence times were inferred using BEAST v2.6.7 based on single-copy orthologous protein-coding gene sequences from 40 species (Kumar et al., 2022a). A total of six secondary calibration nodes were incorporated in this study. For each node, the calibration source, time range, median age and prior distribution are listed below: (1). *Alsophila spinulosa* (NC012818) — *Austroblechnum melanocaulon* (NC035840): secondary calibration, 95% CI = 154.7–228.8 MYA, median = 211 MYA, normal prior distribution. (2) *Austroblechnum melanocaulon* (NC035840) — *Histiopteris incisa* (NC040220): secondary calibration, 95% CI = 52.6–162.5 MYA, median = 125 MYA, normal prior distribution. (3) *Austroblechnum melanocaulon* (NC035840) — *Haplopteris ensiformis* (NC065981): secondary calibration, 95% CI = 136.8–167.0 MYA, median = 152 MYA, normal prior distribution. (4) *Austroblechnum melanocaulon* (NC035840) — *Odontosoria chinensis* (MG913608): secondary calibration, 95% CI = 153.0–228.3 MYA, median = 180 MYA, normal prior distribution. (5) *Austroblechnum melanocaulon* (NC035840) — *Hypodematium crenatum* (NC035857): secondary calibration, 95% CI = 73.2–140.5 MYA, median = 107 MYA, normal prior distribution. (6) *Cystopteris chinensis* (NC035843) — *Asplenium nidus* (NC045119): secondary calibration, 95% CI = 67.3–134.9 MYA, median = 103 MYA, normal prior distribution. All secondary calibration ages were retrieved from TimeTree database.

A strict molecular clock model was applied, and Markov chain Monte Carlo analysis was run for 10,000,000 generations. Tree parameters were sampled every 1,000 generations, and the first 25% of the sampled trees were discarded as burn-in. The remaining trees were combined to generate a consensus tree and estimate node-based divergence times. Tracer v1.7.1 was used to evaluate the BEAST output, with effective sample size (ESS) values exceeding 200 considered to indicate adequate sampling (Rambaut et al., 2018). Finally, FigTree v1.4.4 was used to visualize and edit the resulting time-calibrated phylogenetic tree. All secondary calibration ages were retrieved from TimeTree database. Divergence time estimation for *Woodwardia* was performed in BEAST with a strict molecular clock. We did not test the rate-constancy assumption, which was adopted to reduce parameter complexity under limited fossil calibrations.

### 2.12 Molecular identification

A custom script was used to convert the annotation file of the reference chloroplast genome of *W. japonica* (NC050356) into the format required by mVISTA (http://genome.lbl.gov/vista/mvista/submit.shtml). The mVISTA program was subsequently executed in the Shuffle-LAGAN mode with the three *Woodwardia* chloroplast genome sequences and the processed annotation file as the input. The resulting plots were manually checked, and overlapping gene labels were adjusted for clarity. In the mVISTA output, the y-axis represents sequence identity between each query and the reference, with the displayed range of 50–100%. Regions exhibiting pronounced fluctuations in the curve indicate relatively high levels of sequence divergence between the sample and the reference.

By using the *W. japonica* (NC050356) chloroplast genome as the reference, the three *Woodwardia* chloroplast genomes were globally aligned using MAFFT v7.429. Nucleotide diversity (Pi) across all the chloroplast genomes was then analyzed using a sliding window approach implemented in DnaSP v6, with a window length of 600 bp and a step size of 200 bp.

Sequences spanning the *rrn16–rps12_exon2* region were extracted from 15 comparative species using Perl scripts. These sequences were aligned with MAFFT v7.429, and regions with ambiguous alignments (typically those containing gaps) were trimmed using Gblocks 0.91b (Castresana, 2000.). The trimmed alignment was used to construct a phylogenetic tree. Because the two candidate regions reside within the inverted repeat (IR) region and are reverse complements of each other, only one copy was retained for tree construction. *Lycopodium japonicum* (NC085262) was selected as the outgroup. ML phylogenetic inference was conducted using IQ-TREE v1.6.1. The best-fitting nucleotide substitution model was selected with ModelFinder implemented in IQ-TREE based on the BIC, which yielded TN+F+R2 as the optimal model. The number of bootstrap replicates was set to 1,000.

## 3 Results

### 3.1 Features of the organelle genomes

The mitochondrial genomes of *W. japonica*, *W. unigemmata*, and *W. prolifera* exhibited complex reticulate structures (**Figure 1D, E, F**). Based on the assembly graph, HiFi reads were used to validate the connections. Then, according to the connections and depth, one of the paths was selected and resolved to obtain the inferred final circular mitochondrial genome sequence (**Supplementary Figure S8**). This circular sequence contains all contigs (87 contigs for the mitochondrial genome of *W. japonica*, 61 for that of *W. unigemmata*, and 78 for that of *W. prolifera*). We mapped the long-read data to the final assembly; the coverage was good, with no breakpoints at any position in the sequence (see **Supplementary Figures S2**-**S7** for coverage plots).

The total lengths of the mitochondrial genome sequences of *W. japonica*, *W. unigemmata*, and *W. prolifera* varied substantially, measuring 485,541 bp, 779,752 bp, and 564,263 bp, respectively, with GC contents of 48.24%, 42.67%, and 48.4%, respectively. The average sequencing coverages were 200.72 ×, 66.87 ×, and 56.48 ×, respectively, and no sequence gaps or structural breakpoints were detected at any position (**Supplementary Figure 9**). Annotation of the mitochondrial genomes of the three *Woodwardia* species revealed that *W. japonica*, *W. unigemmata*, and *W. prolifera* contained 151, 306, and 226 genes, respectively (**Figure 2, H, I**). Each mitochondrial genome included one pseudogene (*rps11*). The distribution of gene types is summarized in **Supplementary Table S1**.

**Figure 2:**
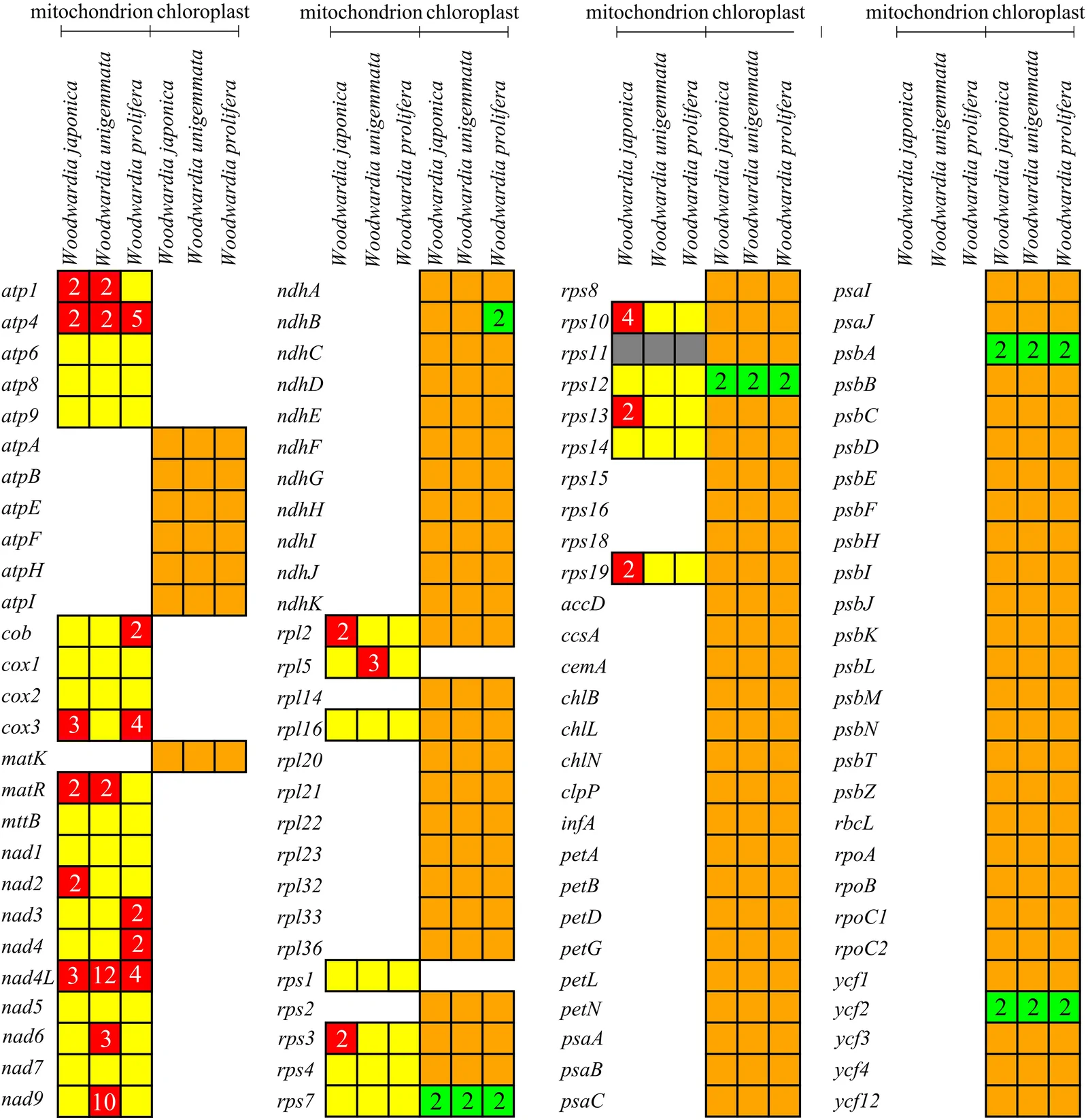
Analysis of the presence and absence of PCGs in the mitochondrial and chloroplast genomes of three *Woodwardia* species. Yellow and orange indicate genes present in the mitochondrial and chloroplast genomes, respectively. Gray represents pseudogenes in the mitochondrial genome. Red and green denote duplicated genes in the mitochondrial and chloroplast genomes, respectively, with numbers indicating the corresponding copy numbers.

The chloroplast genome of *W. prolifera* is 155,328 bp in length with an average sequencing depth of 1,033.36 × (**Supplementary Figure 9**) and encodes a total of 132 genes, including 89 PCGs, 8 rRNA genes, and 35 tRNA genes (**Figure 1**). Among these genes, *rps16*, *trnG-UCC*, *atpF*, *rpoC1*, *trnL-UAA*, *trnV-UAC*, *petB*, *petD*, *rpl16*, *rpl2*, *trnT-UGU*, *trnI-GAU*, *trnA-UGC*, and *ndhA* each contain a single intron, whereas *ycf3* and *clpP* contain two introns. The *rps12* and *ndhB* genes exhibited trans-splicing events. The chloroplast genomes of the three *Woodwardia* species were highly conserved, showing minimal variation in genome size, GC content, and gene composition in both number and functional category. Comprehensive annotations of mitochondrial and chloroplast genes are provided in **Supplementary Table S2**.

In the mitochondrial genomes, most PCGs were conserved, particularly those encoding subunits of NADH dehydrogenase and cytochrome *c* reductase. In contrast, ribosomal protein genes and succinate dehydrogenase genes exhibited higher variability. The genes *rpl6*, *rpl10*, *rps2*, *rps5*, *rps8*, *rps19*, *sdh3*, and *sdh4* were absent in the mitochondrial genomes of all three species, while each retained a single pseudogene, *rps11*. Regarding gene copy number, *W. unigemmata* showed the highest number of copies, totaling 59 PCGs. Importantly, this count represents total gene copies, not the number of non-redundant unique PCGs. Notably, *nad4L* and *nad9* displayed exceptionally high copy numbers (12 and 10 copies, respectively), which far exceeded those observed in the other two species. These multiple copies are associated with repeated genomic regions rather than independent gene duplication events. In *W. japonica*, multiple copies arising from repeated genomic regions were primarily found among ribosomal protein genes, whereas in *W. prolifera*, such high-copy genes were more prevalent among NADH dehydrogenase-associated genes (**Figure 2**).

In contrast, the gene composition of the chloroplast genomes was more stable. Most photosynthesis-related genes were present as single copies, and all three species contained two copies of *rps12* and *psbA*. Additionally, except for *W. prolifera*, which harbored two copies of *ndhB*, the number and types of non-redundant chloroplast genes were consistent across the three species.

### 3.2 Analysis of selective evolutionary pressure on PCGs

In the mitochondrial genes of the three *Woodwardia* species, the average Ka/Ks value <1 of 26 PCGs, except for *atp6*, *mttB*, *nad2*, *rps3*, and *rps4*. This finding indicates that these 26 PCGs have predominantly undergone purifying selection during evolution and have maintained relatively stable protein functions. Additionally, the Ka/Ks value >1 of *rps1* between *W. japonica* and *W. prolifera*. In the chloroplast genomes of the three *Woodwardia* species, the average Ka/Ks value <1 of the 84 shared PCGs, suggesting that these genes also underwent purifying selection and maintained stable protein functions. However, the Ka/Ks value >1 between *W. japonica* and *W. unigemmata* for *psbE* and *cemA*. For *psbL*, the value >1 between *W. japonica* and *W. prolifera* (**Figure 3**).

**Figure 3:**
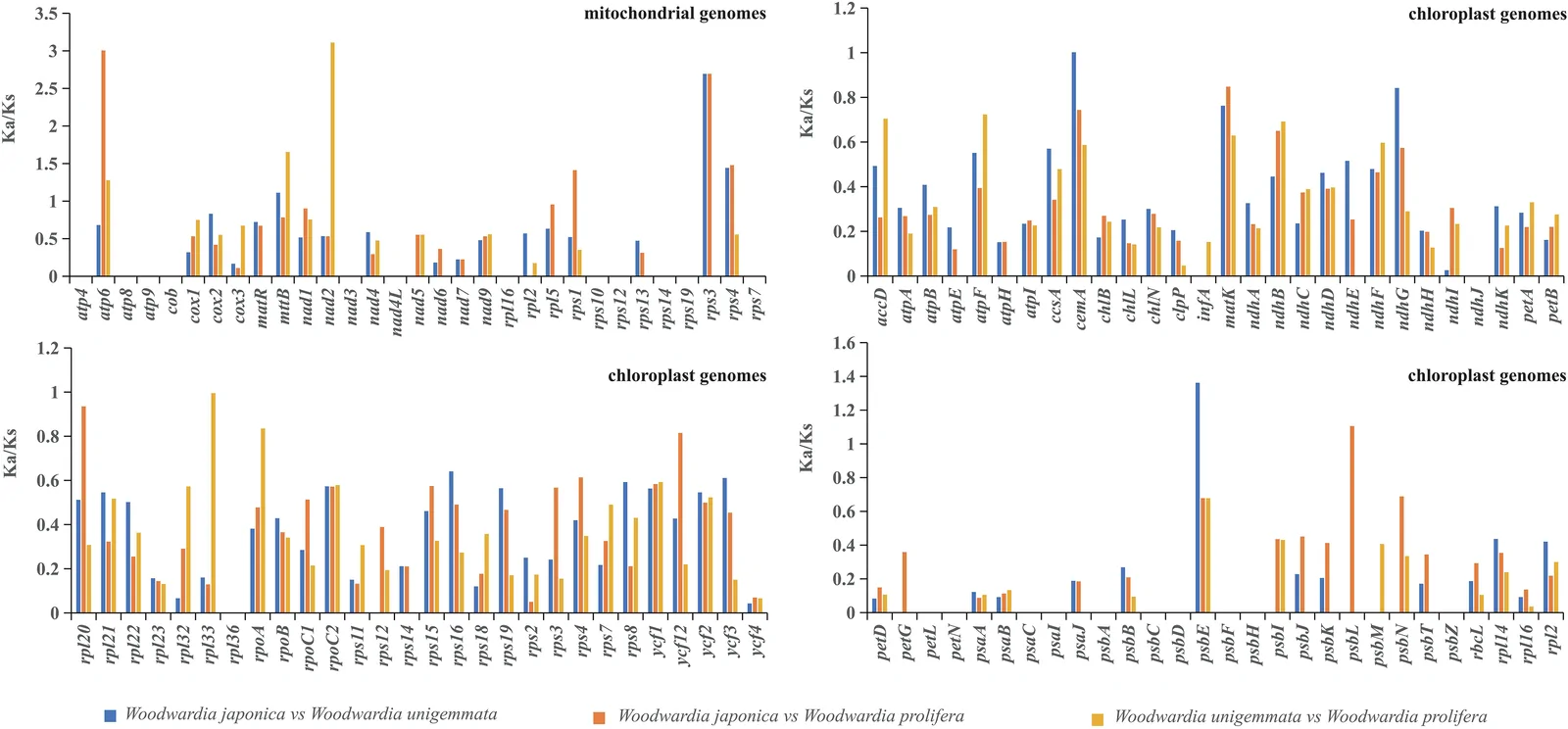
Ka/Ks ratio analysis of PCGs in the mitochondrial and chloroplast genomes of three *Woodwardia* species.

### 3.3 Codon usage analysis

The mitochondrial genomes of *W. japonica*, *W. unigemmata*, and *W. prolifera* contained 8,404, 8,325, and 8,376 codons, respectively (**Figure 4**). Except for the start codon (AUG), leucine (CUU), and tryptophan (UGG) in *W. japonica* and *W. prolifera*, and the start codon (AUG), threonine (ACC), and tryptophan (UGG) in *W. unigemmata*, which had RSCU values of 1, the mitochondrial PCGs exhibited a general codon usage bias. Among the 31 shared PCGs in the mitochondrial genomes of the three *Woodwardia* species, leucine showed a strong preference for the UUA codon, which had the highest RSCU value among all mitochondrial PCGs.

**Figure 4:**
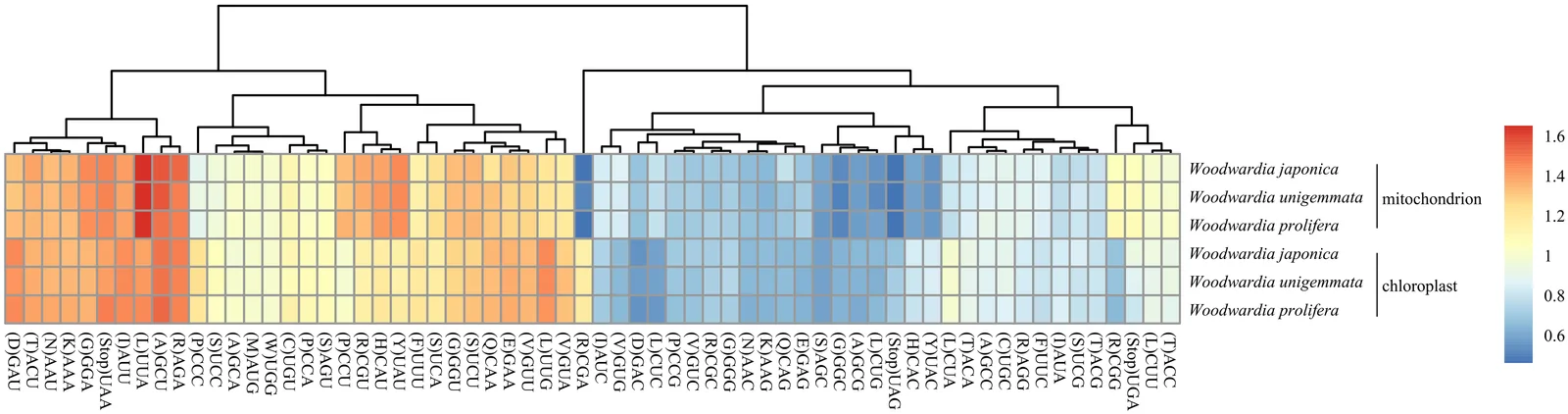
Analysis of codon usage bias in the mitochondrial and chloroplast genomes of three *Woodwardia* species.

In the chloroplast genomes, *W. japonica*, *W. unigemmata*, and *W. prolifera* contained 24,100, 24,091, and 24,155 codons, respectively. In *W. japonica*, all codons, except for the start codon (AUG), leucine (CUA), proline (CCU), and tryptophan (UGG), had RSCU values of 1. In *W. prolifera*, all codons, except for the start codon (AUG), leucine (CUA), alanine (GCA), and tryptophan (UGG), had RSCU values of 1. In *W. unigemmata*, all codons, except for the start codon (AUG) and tryptophan (UGG), had RSCU values of 1. These results indicate that the chloroplast PCGs also exhibited a general codon usage bias. Among the 84 shared PCGs in the chloroplast genomes of the three *Woodwardia* species, alanine showed a strong preference for the GCU codon, which had the highest RSCU value among all chloroplast PCGs.

For both mitochondrial and chloroplast genomes, the high-frequency codons (RSCU > 1) of the three *Woodwardia* species predominantly ended with A or U at the third codon position, whereas low-frequency codons (RSCU < 1) mostly ended with G or C.

### 3.4 Analysis of repeat sequences

In the mitochondrial genomes, *W. unigemmata* contained the largest number of SSRs, followed by *W. prolifera* and *W. japonica*, with 260, 197, and 170 SSRs, respectively. Tetranucleotide and pentanucleotide repeats were the predominant types across all three species. In contrast, the chloroplast genomes exhibited substantially fewer SSRs than the mitochondrial genomes. Specifically, *W. japonica* contained 68 SSRs, whereas both *W. unigemmata* and *W. prolifera* had 57 SSRs each. Chloroplast SSRs of all three species predominantly contained mononucleotide repeats, and tetra- or pentanucleotide repeats were absent (**Figure 5A**).

**Figure 5:**
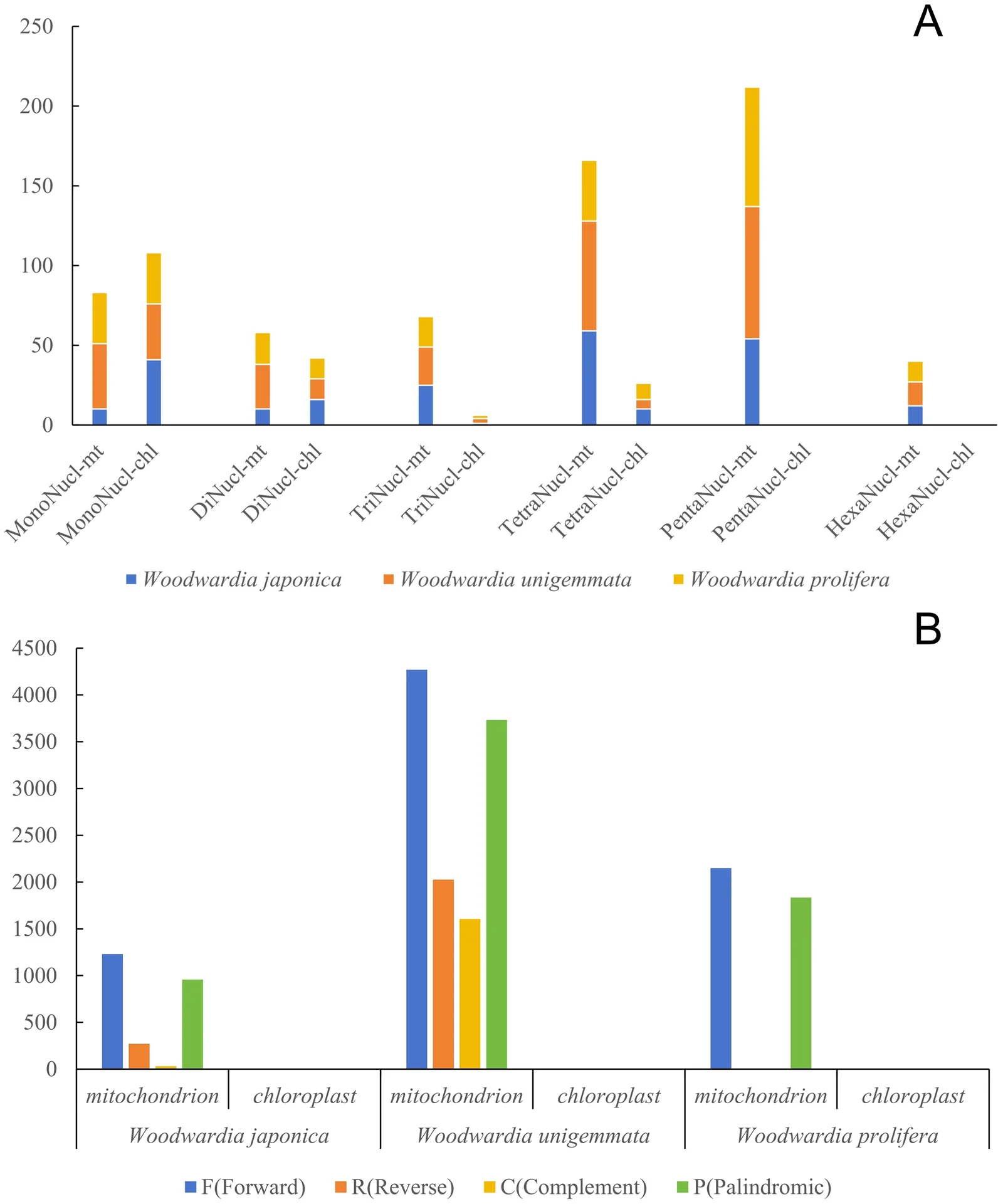
SSR and tandem repeat sequence analyses of mitochondrial and chloroplast genomes in three *Woodwardia* species. **(A)** SSR motif distribution; **(B)** Types and counts of tandem repeats.

Four types of long repeats—forward, reverse, palindromic, and complementary—were identified in the mitochondrial genomes of all three *Woodwardia* species. *W. unigemmata* displayed the highest number of tandem repeats (11,632), significantly exceeding those in *W. japonica* (2491) and *W. prolifera* (3995). Forward and palindromic repeats were the most prevalent types in all species. In the chloroplast genomes, however, reverse and complementary repeats were undetected in all three species. Additionally, the numbers of forward and palindromic repeats were markedly lower in the chloroplast genomes than in the mitochondrial genomes, with each species containing fewer than eight repeats (**Figure 5B**).

### 3.5 Analysis of RNA editing sites

In the mitochondrial genomes of the three *Woodwardia* species, a total of 812 RNA editing sites were predicted in the PCGs of *W. japonica*, representing the highest number among the three species. Both *W. unigemmata* and *W. prolifera* exhibited 783 predicted RNA editing sites each. Compared to these two species, *W. japonica* showed a similar number of predicted editing sites across most genes, except for *atp6*, which contained substantially more sites in *W. japonica* (31) than in *W. unigemmata* (16) and *W. prolifera* (16). Among all mitochondrial PCGs, *nad4* (80–81 sites), *cox1* (78–80 sites), and *nad5* (70–71 sites) harbored the highest numbers of predicted RNA editing sites. Most of these editing sites occurred at the second codon position, with the predominant amino acid change being proline-to-leucine.

In the chloroplast genomes, RNA editing sites were more abundant. A total of 1,505, 1,487, and 1,427 RNA editing sites were predicted in the PCGs of *W. japonica*, *W. unigemmata*, and *W. prolifera*, respectively. Notably, no RNA editing sites were predicted in *psbI* of *W. unigemmata* or *psbT* of *W. prolifera*. Among the chloroplast PCGs, *ycf2* (160–162 sites), *ycf1* (131–141 sites), and *rpoC2* (114–115 sites) contained the largest number of predicted editing sites. As observed in the mitochondrial genomes, most RNA editing sites occurred at the second codon position, with serine-to-leucine being the most frequent amino acid conversion (**Figure 6**).

**Figure 6:**
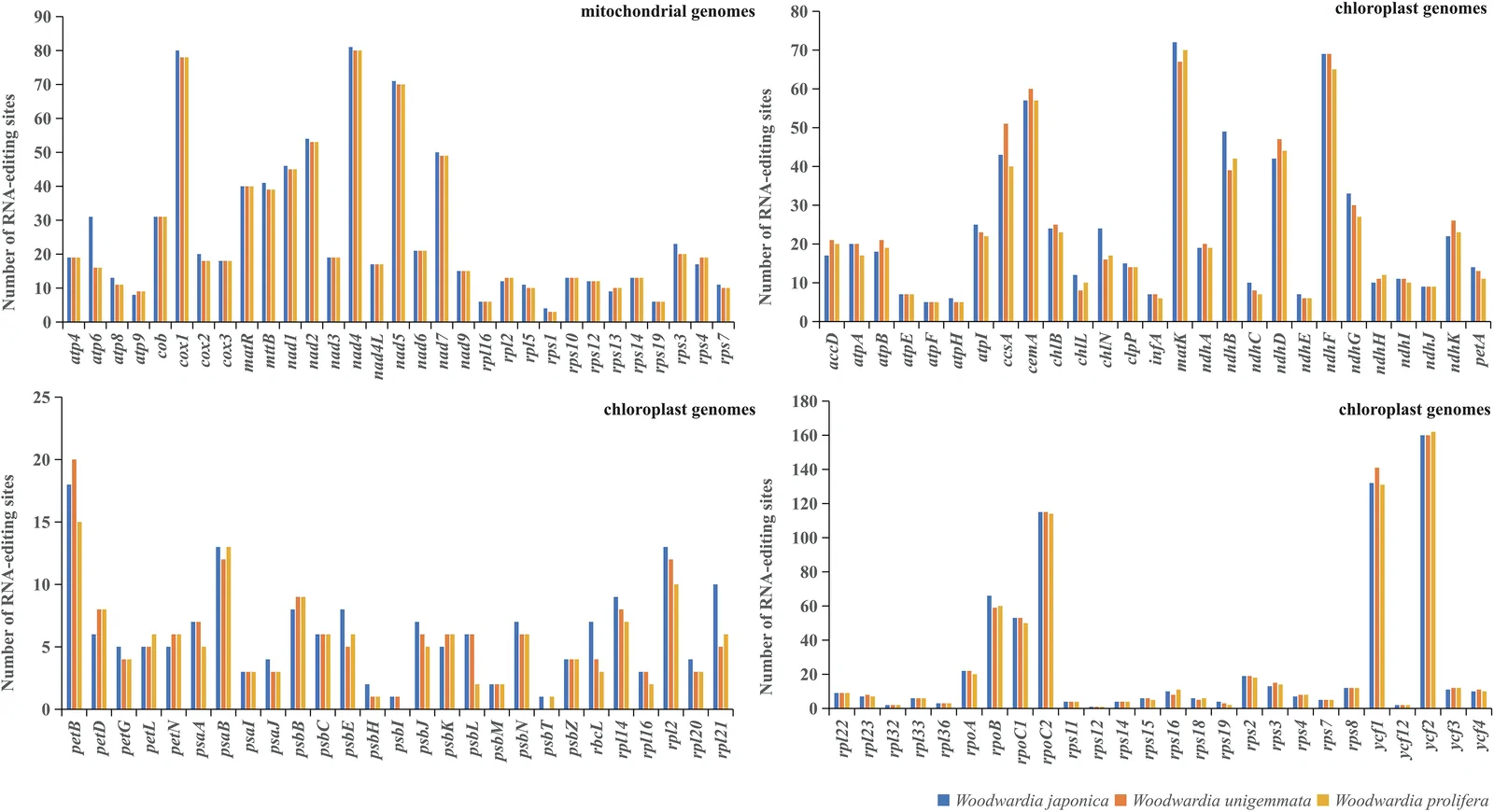
Prediction and analysis of RNA editing sites in the mitochondrial and chloroplast genomes of three *Woodwardia* species.

### 3.6 Synteny analysis

In the mitochondrial genomes, 1,067 and 1,630 syntenic blocks exceeding 1,000 bp were identified between *W. japonica* and *W. unigemmata*, and between *W. unigemmata* and *W. prolifera*, respectively. The total lengths of syntenic blocks among the three *Woodwardia* mitochondrial genomes were relatively large. However, the genomic arrangements of these syntenic blocks varied significantly among the species, suggesting that the mitochondrial genomes of *Woodwardia* are structurally highly divergent. In contrast, the chloroplast genomes contained only 10 and 20 syntenic blocks longer than 1,000 bp between *W. japonica* and *W. unigemmata*, and between *W. unigemmata* and *W. prolifera*, respectively. These findings indicate a markedly greater level of structural conservation in the chloroplast genomes than in the mitochondrial genomes (**Figure 7**).

**Figure 7:**
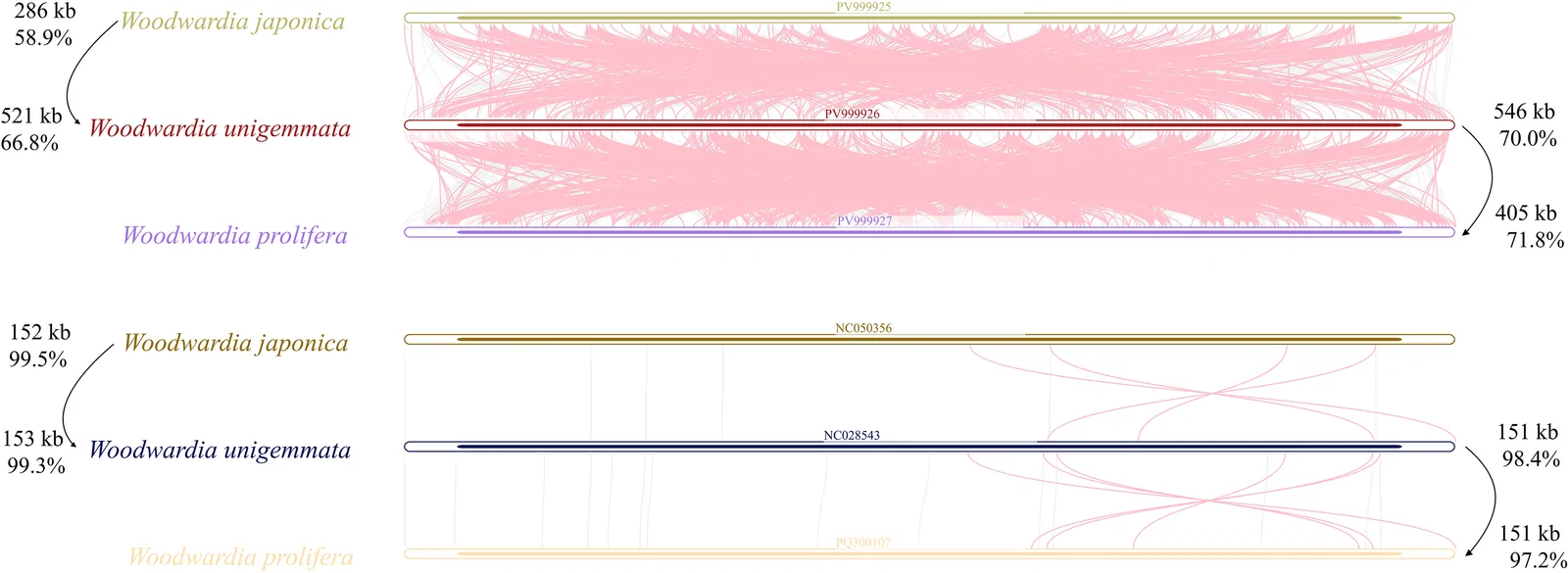
Synteny analysis of the mitochondrial and chloroplast genomes of three *Woodwardia* species. Arcs in the figure connect homologous genomic regions. Red arcs represent sequences in reverse orientation, whereas gray arcs denote sequences in forward orientation. Regions lacking syntenic blocks correspond to sequences unique to a given species.

### 3.7 Identification of MTPTs

Based on nucleotide sequence similarity, 15 (including two duplicated fragments), 4 (including one duplicated fragment), and 9 (including three duplicated) putative MTPTs were identified in the mitochondrial genomes of *Woodwardia japonica*, *W. unigemmata*, and *W. prolifera*, respectively (**Figure 8**). Among these, two, one, and three fragments were duplicated in *W. japonica*, *W. unigemmata*, and *W. prolifera*, respectively. The total lengths of the long homologous fragments exceeding 1,000 bp were 24,757 bp (22,493 bp after duplicate removal) in *W. japonica*, 6,891 bp (5,754 bp after duplicate removal) in *W. unigemmata*, and 22,076 bp (15,553 bp after duplicate removal) in *W. prolifera*. The longest individual fragments in the three species measured 3,777 bp, 3,343 bp, and 5,963 bp, respectively.

**Figure 8:**
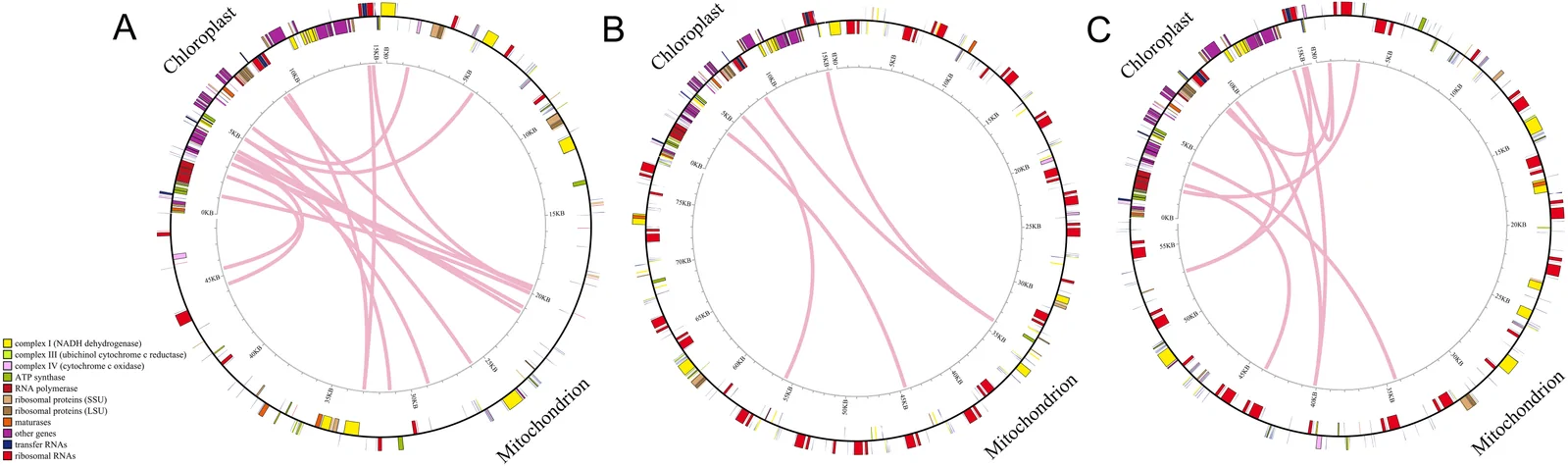
Transfer events of chloroplast genome from three *Woodwardia* species to mitochondrial genome. **(A)** *W.japonica*; **(B)** *W. unigemmata*; **(C)** *W. prolifera*. Lines connecting the arcs indicate homologous genomic regions greater than 1,000 bp in length between the chloroplast and mitochondrial genomes.

Annotation of these homologous regions revealed the presence of several intact genes within the long fragments. Specifically, *W. japonica* contained three PCGs (*ycf3*, *psbD*, and *petA*), while *W. prolifera* contained two PCGs (*psaA* and *ycf3*) and two tRNA genes (*trnS-GGA* and *trnA-UGC*). In contrast, no complete genes were identified in the long homologous fragments of *W. unigemmata*.

### 3.8 Phylogenetic and divergence time analyses

Phylogenetic trees were constructed using homologous single-copy genes from both mitochondrial and chloroplast genomes (**Figure 9**). The topological structures of the two trees were identical, with the three *Woodwardia* species forming a single monophyletic clade. *W. unigemmata* and *W. prolifera* are the most closely related; the clade formed by these two species is sister to *W. japonica*. All nodes in both phylogenetic trees exhibited high bootstrap support, indicating robust reliability. The chloroplast genome-based phylogenetic tree displayed marginally higher bootstrap support across most branches compared to the mitochondrial genome-based tree. Time-calibrated phylogenetic reconstruction (**Figure 10**) confirmed that the three species of *Woodwardia* form a monophyletic clade. *W. japonica* diverged first from their common ancestor at approximately 15.23 Ma, followed by interspecific divergence between the more closely related *W. unigemmata* and *W. prolifera* at about 10.58 Ma.

**Figure 9:**
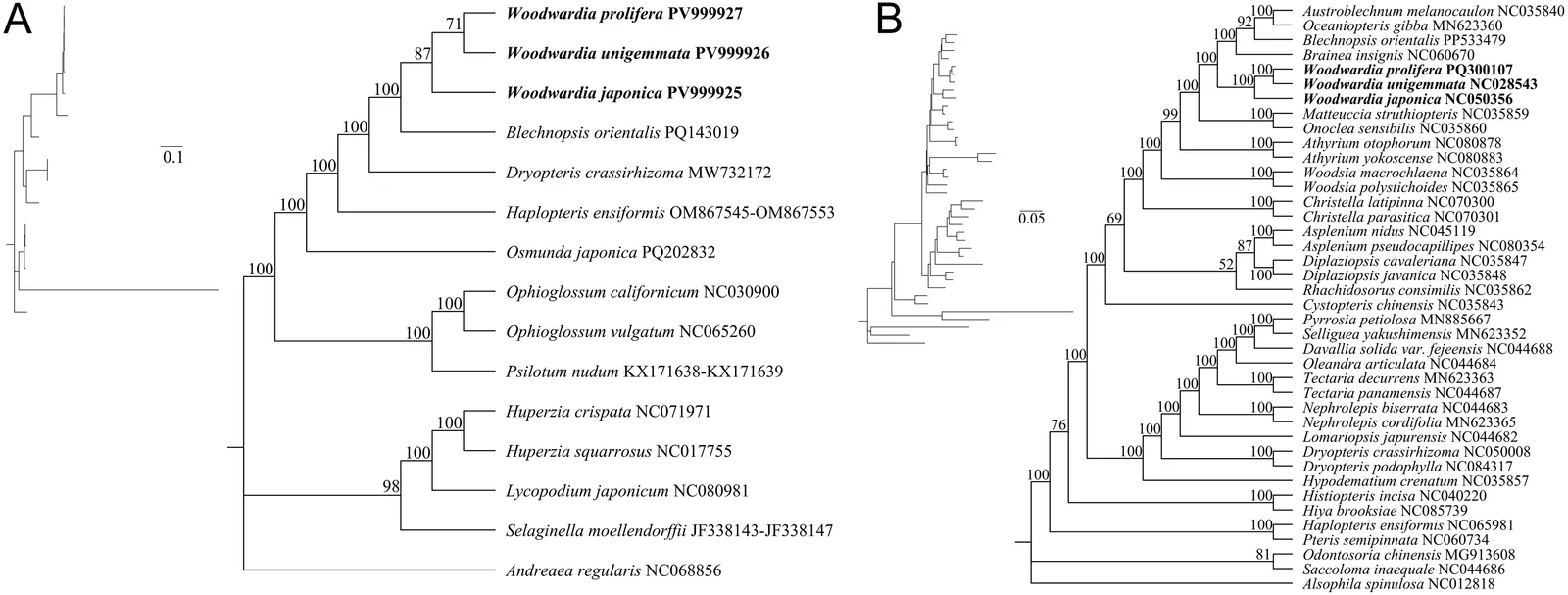
Maximum-likelihood phylogenetic trees of three *Woodwardia* species inferred from mitochondrial and chloroplast genomes. **(A)** Phylogenetic tree inferred from the mitochondrial genome; **(B)** Phylogenetic tree inferred from the chloroplast genome.

**Figure 10:**
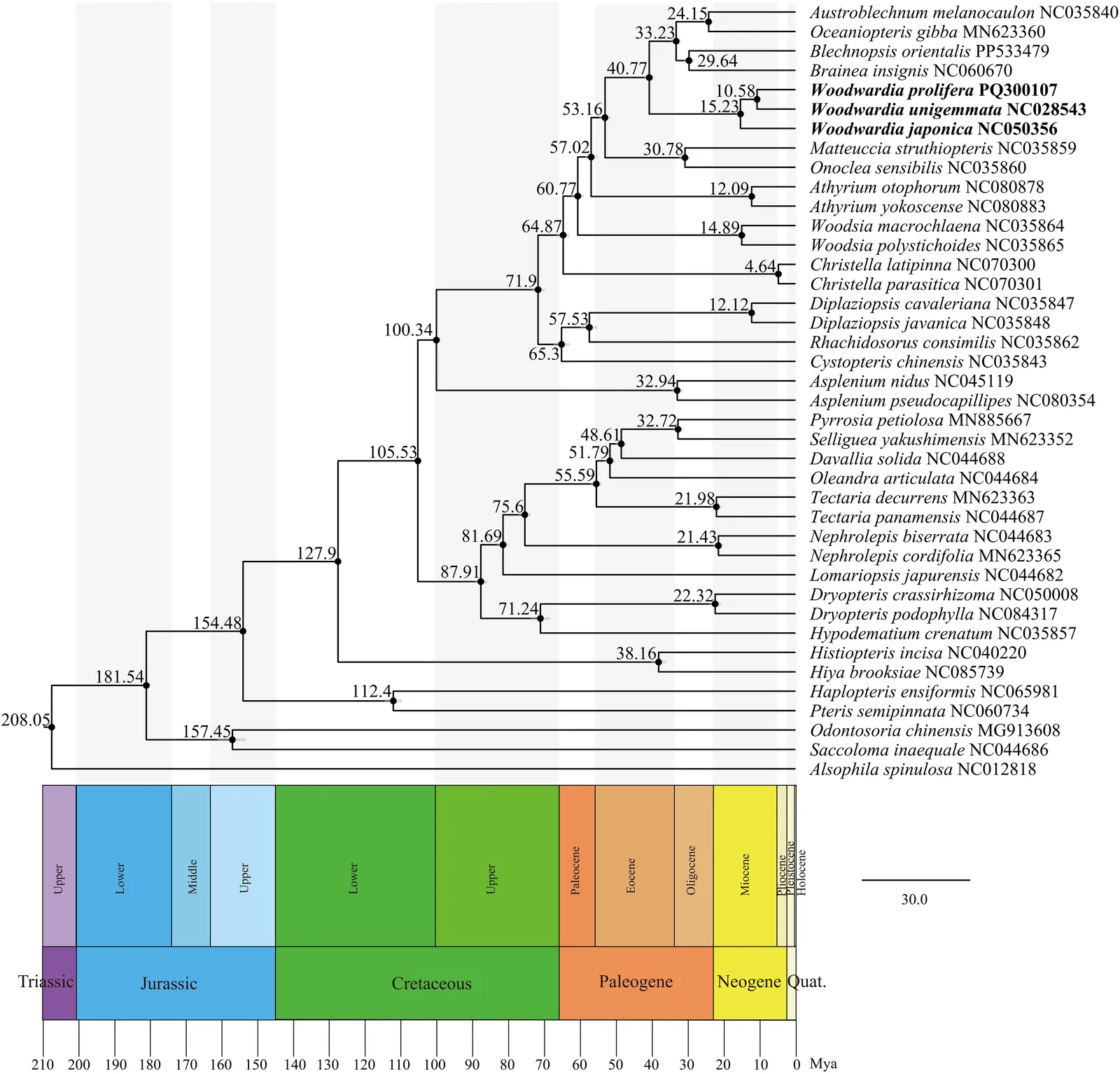
Phylogenetic tree of evolutionary divergence times for three *Woodwardia* species.

### 3.9 Molecular identification

Comparative analysis using mVISTA (**Figure 11A**) revealed that the noncoding regions among the three *Woodwardia* species exhibited greater nucleotide divergence than the coding regions. The most variable regions were *rrn16–rps12_exon2* and *rps12_exon2–rrn16*. The Pi values ranged from 0 to 0.22556, with the highest values observed in *rrn16–rps12_exon2* and *rps12_exon2–rrn16*, consistent with the mVISTA results (**Figure 11B**).

**Figure 11:**
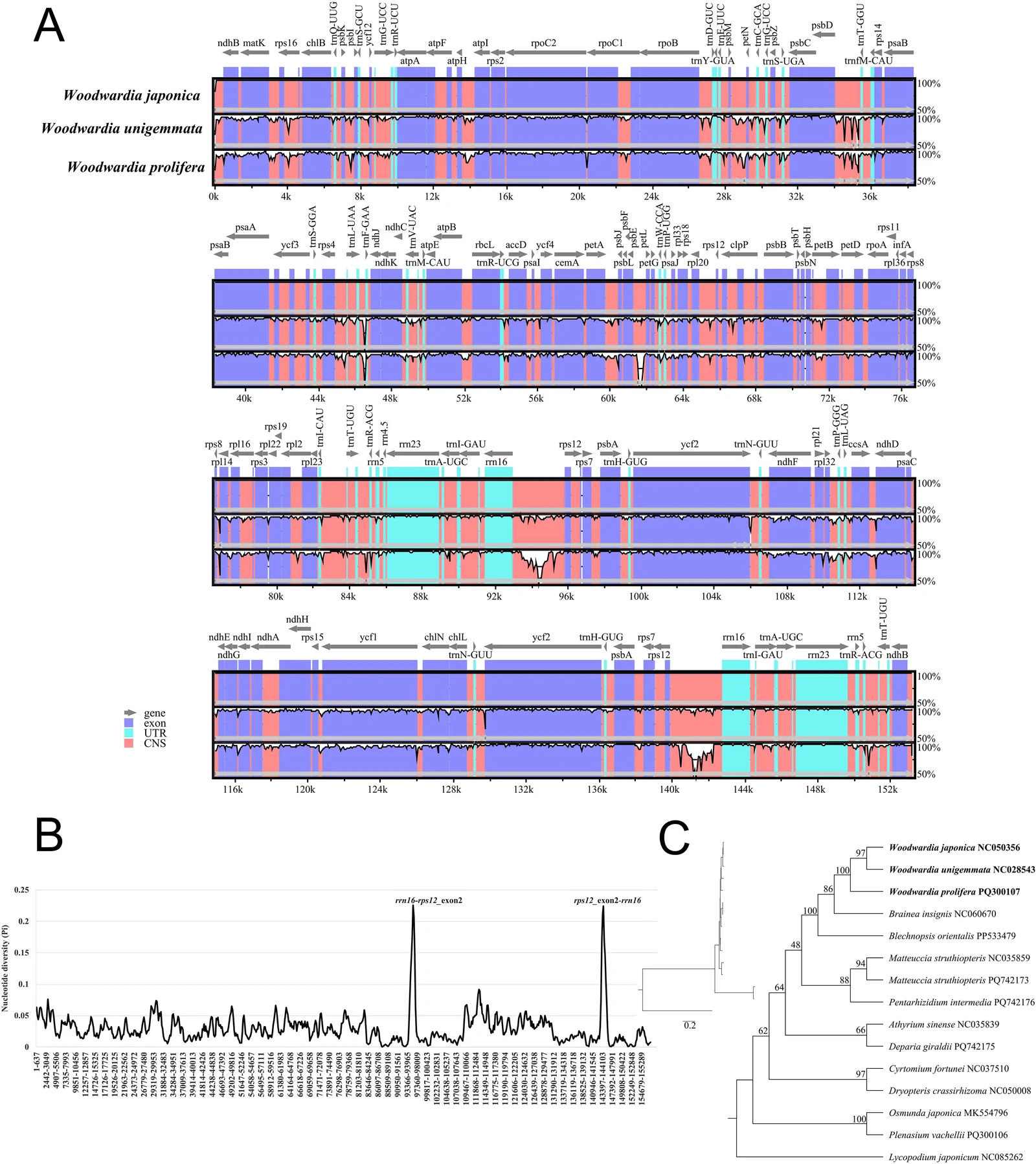
Comparative analyses for molecular identification of three *Woodwardia* species: **(A)** Sequence identity and divergence of chloroplast genomes; **(B)** Nucleotide diversity (Pi) analysis of chloroplast genes; **(C)** Phylogenetic reconstruction based on the highly variable regions *rrn16–rps12_exon2/rps12_exon2–rrn16*.

Phylogenetic analysis based on the chloroplast *rrn16–rps12_exon2* and *rps12_exon2–rrn16* fragments (**Figure 11C**) showed that each of the three *Woodwardia* species formed distinct monophyletic clades, clearly separated from the outgroup. The tree topology was stable, with key nodes supported by high bootstrap values (mostly high, with the lowest at a moderate-to-high level), indicating that these regions possess excellent resolution at the species level. Within this topology, *W. unigemmata* and *W. prolifera* formed a sister clade, which together with *W. japonica* constituted a well-supported larger branch. Overall, these findings demonstrate that the *rrn16–rps12_exon2* and *rps12_exon2–rrn16* regions are potentially effective molecular markers for species identification within the genus *Woodwardia*.

## 4 Discussion

### 4.1 Characteristics of organelle genomes in three medicinal *Woodwardia* species

The mitochondrial genomes of the three *Woodwardia* species exhibited complex reticulate and dynamic structures, consistent with previous reports on the mitochondrial genome architecture of *Woodwardia* species in the family Blechnaceae (Huang et al., 2025). Notably, substantial differences in mitochondrial genome length and gene number among the three species indicate that extensive structural variation can occur even within congeners. In contrast, the chloroplast genomes of the three *Woodwardia* species are markedly more conserved. Specifically, the chloroplast genome of *W. prolifera* closely resembles those of the other two species in both size and GC content; moreover, it structurally retains the typical quadripartite organization observed in most fern chloroplast genomes (Huang et al., 2024).

In the mitochondrial genomes, several ribosomal protein genes (e.g., *rpl6* and *rpl10*) and succinate dehydrogenase genes (*sdh3* and *sdh4*) were absent in all three species, and *rps11* occurred as a pseudogene. Previous studies suggest that many mitochondrial genes may have been transferred to the nuclear genome or functionally replaced by nuclear genes during evolution (Butenko et al., 2024). Fern mitochondrial genomes are known to undergo varying degrees of gene loss and recombination within certain gene families (Sun et al., 2024a), and the current findings further support this evolutionary trend. With respect to gene copy number variation, *W. unigemmata* exhibited a markedly higher number of mitochondrial gene copies than *W. japonica* and *W. prolifera*. It should be noted that these high counts refer to total gene copies derived from repeated genomic regions, rather than an increased number of non-redundant unique mitochondrial genes. In *W. japonica*, genes with high copy counts were predominantly ribosomal protein genes, whereas in *W. prolifera*, they were enriched in NADH dehydrogenase genes, indicating divergent patterns of copy accumulation from genomic repeats across the three *Woodwardia* species. A previous study proposed that when selective pressure on genes is relaxed or the effective population size is reduced, gene transfer and copy number variation may occur in bursts (Butenko et al., 2024). Therefore, the divergence in copy number patterns among *Woodwardia* species likely reflects different modes of mitochondrial genome restructuring, where repeated genomic regions generate variable numbers of homologous gene copies.

In contrast, the chloroplast genomes of the three species show a high degree of conservation, aligning with prior observations that fern chloroplast genomes are highly stable in non-redundant gene content, with variations primarily originating from the expansion of the inverted repeat regions or structural rearrangements rather than gene loss or changes in copy number (Wolf and Roper, 2008).

Among the three *Woodwardia* species, *W. unigemmata* possesses the longest mitochondrial genome, with the highest number of SSRs, and a substantially greater abundance of long tandem repeats than the other two species. These features suggest that its mitochondrial genome has undergone more active repeat-mediated amplification and structural reorganization. In ferns, large and repeat-rich mitochondrial genomes have been reported in species such as *Ophioglossum californicum* and *Psilotum nudum*, which contain abundant forward and palindromic repeats (Guo et al., 2017). Unlike the mitochondrial genomes, the chloroplast genomes of the three *Woodwardia* species contained substantially fewer repeats, with SSR counts significantly lower than those in the mitochondrial genomes and dominated by mononucleotide repeats. No tetra- or pentanucleotide repeats were detected, and only a limited number of forward and palindromic repeats were identified, with no reverse or complementary repeats.

The three species also differed markedly in the number and length of putative MTPTs and in the presence of intact gene sequences within these homologous fragments. Although *W. unigemmata* has the fewest putative MTPT fragments (n = 4), its longest fragment spans 5,963 bp, which represents a substantial insertion. In *W. japonica* and *W. prolifera*, both the total length of putative MTPTs and the number of fragments containing intact gene sequences were higher, suggesting either more frequent retention of plastid-derived sequences or better preservation of the inserted fragments. The presence of intact gene sequences such as *ycf3*, *psbD*, *petA*, and *psaA* suggests that some of these regions are not merely degraded remnants; however, whether they are transcribed, translated, or functional in the mitochondrial genome remains unknown. Prior research has noted that certain plastid-derived mitochondrial sequences can interfere with DNA barcoding and lead to misidentification in applied contexts (Park et al., 2020).

### 4.2 Evolutionary characteristics of organelle genomes in three medicinal *Woodwardia* species

Ka/Ks analysis is a valuable approach for understanding species adaptation, selective pressures, adaptive potential, and patterns of genetic and evolutionary change (Xie et al., 2019). In all three *Woodwardia* species, most PCGs in both mitochondrial and chloroplast genomes were under strong purifying selection. This finding aligns with the essential roles of these two organelles in respiration and photosynthesis (Huang et al., 2025; Singh et al., 2022) and further supports the observation that most organellar PCGs in ferns are subject to negative selection (Sun et al., 2024b). It is worth noting that, at present, studies on selection pressure analysis of mitochondrial protein-coding genes in ferns are still relatively limited, and no cases of Ka/Ks > 1 in *atp6*, *mttB*, *nad2*, *rps3*, and *rps4* have yet been found in other reported fern mitochondrial genomes. However, in angiosperms, such as *Chimonanthus salicifolius* and *Chimonanthus praecox*, mitochondrial genes such as *rps4* have been detected with Ka/Ks > 1 (Fang et al., 2026). Limited by the currently limited sample size of fern mitochondrial genome studies, the above genes were detected with Ka/Ks > 1 in the three *Woodwardia* species in this study, which may reflect that these genes have experienced relaxed selective constraints in the *Woodwardia* mitochondrial genome. Among chloroplast genes, this study detected Ka/Ks > 1 for *psbE* and *cemA* in the pairwise comparison between *W. japonica* and *W. unigemmata*, and Ka/Ks > 1 for *psbL* in the pairwise comparison between *W. japonica* and *W. prolifera*. In published studies on selection pressure in fern chloroplast genomes, *psbL* has been reported to show ω > 1 in epiphytic groups of Pteridaceae, interpreted as relaxed selective constraints (Peng et al., 2024); *psbE* has only been detected with a few selected amino acid sites in Cyatheaceae studies (Peng et al., 2024); and *cemA* has not yet been reported with Ka/Ks > 1 in ferns, with abnormal selection pressure signals of this gene mostly found in angiosperm groups (e.g., *Saxifraga*) (Li et al., 2025).

Codon usage bias provides valuable insights into genome evolution from a molecular evolutionary perspective (Hershberg and Petrov, 2008). In mitochondrial PCGs, all 31 shared genes among the three *Woodwardia* species showed a preference for the UUA codon (encoding leucine), indicating that UUA is a dominant high-frequency codon in their mitochondrial genomes. In chloroplast PCGs, all 84 shared genes exhibited a preference for the GCU codon (encoding alanine). Among high-frequency codons (RSCU > 1), the third nucleotide position was predominantly occupied by A or U, whereas in low-frequency codons (RSCU < 1), G or C mostly occurred in the third position. This pattern matches the previously reported A/U-ending codon preference observed for mitochondrial codon usage in other species of the family Blechnaceae (Huang et al., 2025), suggesting conserved mutational or translational selection pressures within the family.

In the mitochondrial genomes of these three *Woodwardia* species, *nad4*, *cox1*, and *nad5* contained the highest densities of RNA editing sites. These genes play central roles in assembling mitochondrial respiratory chain complexes and facilitating electron transport, and they are highly conserved in function (Hao et al., 2021). Most editing events occurred at the second codon position, with proline-to-leucine being the most frequent amino acid substitution, a trend consistent with prior findings (Lukeš et al., 2021). In ferns, mitochondrial RNA editing is not only extensive but also includes both C-to-U and U-to-C types. In contrast, the U-to-C type has been largely lost in angiosperms (Knoop, 2023).

In chloroplast PCGs, the number of predicted RNA editing events in the three *Woodwardia* species exceeded those in the mitochondrial genomes. Similar to the mitochondria, chloroplast RNA editing primarily occurred at the second codon position, with serine-to-leucine being the most common amino acid conversion. Such conversions frequently occur in genes encoding membrane-associated proteins or subunits of photosynthetic complexes and may enhance the hydrophobicity and structural stability of photosynthetic membrane proteins (Lukeš et al., 2021).

Plant mitochondrial genomes are typically enriched in repetitive sequences and are prone to undergo homologous recombination, resulting in highly dynamic genome architectures (Arrieta-Montiel and Mackenzie, 2011). In the present study, the syntenic arrangement among the mitochondrial genomes of the three *Woodwardia* species varied significantly, indicating extensive structural rearrangements since their divergence within the genus. In contrast, the chloroplast genomes of the three species were structurally highly conserved, consistent with the general pattern of chloroplast genome evolution across land plants (Cauz-Santos, 2025). Overall, our results indicate that the chloroplast genomes of *Woodwardia* are structurally stable and exhibit only limited small-scale variation, supporting the established evolutionary model in which chloroplast genomes are relatively conserved while mitochondrial genomes display high plasticity among plant organelle genomes.

### 4.3 Phylogenetic relationships and molecular identification of the three medicinal *Woodwardia* species

In all phylogenetic trees, *W. unigemmata* and *W. prolifera* consistently formed a sister clade, which together showed a sister relationship with *W. japonica*. The chloroplast genome-based phylogenetic tree exhibited higher bootstrap support values than the mitochondrial genome-based tree; this finding was consistent with prior studies indicating that chloroplast genomes often yield greater resolution for plant phylogenetic reconstruction, particularly during the recent intra-generic divergence (Kumar et al., 2022b). Furthermore, the intergeneric and interfamilial relationships reconstructed in this study align with the PPG I classification system (The Pteridophyte Phylogeny Group, 2016). These results reinforce the reliability of organelle genome-based phylogenies in resolving branching relationships and provide molecular support for fern classification at the family level.

However, because of the extreme scarcity of publicly available mitochondrial genome data for ferns—only a little more than 10 species are currently represented in the NCBI database (Wang et al., 2024)—the mitochondrial genome-based phylogenetic analysis could not fully elucidate the relationships between *Woodwardia* and other fern lineages. This limitation highlights the urgent need for expanded mitochondrial genome sequencing across fern taxa. Time-calibrated phylogenetic analyses indicated that divergence among the three *Woodwardia* species occurred during the Miocene epoch. This finding is consistent with other phylogenetic studies on ferns, which suggest that the Miocene may have been a period of accelerated diversification for multiple fern genera (Cárdenas and Lehtonen, 2023; Testo and Sundue, 2016). Divergence time estimates clarify the evolutionary history of *Woodwardia* during the Miocene and establish a critical temporal framework for interpreting its current geographic distribution and ecological adaptation. These divergence time results corroborate the organelle genome-based phylogenetic analyses by confirming that *W. unigemmata* and *W. prolifera* are more closely related to each other than either of them to *W. japonica*. Nevertheless, the limitation of this dating analysis is the untested constant-rate assumption of the strict molecular clock, and relaxed clock models with expanded sampling should be used in future work to validate our *Woodwardia* divergence times.

Multiple studies have demonstrated that specific regions of the chloroplast genome can provide high discriminatory power in identifying medicinal plants, even among morphologically similar or closely related species within the same genus (Chen et al., 2023b). The phylogenetic tree constructed using the highly variable *rrn16–rps12_exon2* and *rps12_exon2–rrn16* fragments from 14 Guan Zhong medicinal materials showed that these regions possess strong resolving power for differentiating the three *Woodwardia* species. Therefore, the *rrn16–rps12_exon2* or *rps12_exon2–rrn16* fragment represents a promising candidate DNA barcode for accurately identifying *W. japonica*, *W. unigemmata*, and *W. prolifera* in Guan Zhong medicinal materials.

## 5 Conclusion

In this study, we successfully completed the sequencing and assembly of the mitochondrial genomes of *W. japonica*, *W. unigemmata*, and *W. prolifera* and the chloroplast genome of *W. prolifera*. Comprehensive analyses were conducted on organelle genome structure, evolutionary characteristics, phylogeny, and molecular identification. Substantial differences were observed in mitochondrial genome size and gene content and organization among the three *Woodwardia* species, whereas the chloroplast genomes remained relatively conserved and highly similar. Phylogenetic analyses based on both organelle genomes and divergence time estimations indicated that *W. unigemmata* and *W. prolifera* are more closely related and diverged more recently. Combined assessments of highly variable regions and phylogenetic relationships revealed that the intergenic spacer *rrn16–rps12_exon2* or *rps12_exon2–rrn16* could effectively distinguish the three Guan Zhong-related *Woodwardia* species, suggesting its potential as a reliable DNA barcode for medicinal material identification. Overall, these findings provide comprehensive insights and detailed, reliable organelle genomic information for the phylogenetic analysis and molecular identification of *W. japonica*, *W. unigemmata*, and *W. prolifera*.

## Data Availability

The complete sequence of the organelle genome is accessible in the GenBank nucleotide database (https://www.ncbi.nlm.nih.gov/nucleotide/) with accession number PV999925-PV999927 and PQ300107.

## References

[B1] Alverson A. J. Wei X. Rice D. W. Stern D. B. Barry K. Palmer J. D. (2010). Insights into the evolution of mitochondrial genome size from complete sequences of *Citrullus lanatus* and *Cucurbita pepo* (Cucurbitaceae). Mol. Biol. Evol. 27, 1436–1448. doi: 10.1093/molbev/msq029 20118192 PMC2877997

[B2] Arrieta-Montiel M. P. Mackenzie S. A. (2011). “ Plant mitochondrial genomes and recombination,” in Plant Mitochondria. Ed. Kempken F. ( Springer Press, New York, NY), 65–82.

[B3] Bankevich A. Nurk S. Antipov D. Gurevich A. A. Dvorkin M. Kulikov A. S. . (2012). SPAdes: A new genome assembly algorithm and its applications to single-cell sequencing. J. Comput. Biol. 19, 455–477. doi: 10.1089/cmb.2012.0021 22506599 PMC3342519

[B4] Bi C. Shen F. Han F. Qu Y. Hou J. Xu K. . (2024). PMAT: An efficient plant mitogenome assembly toolkit using low-coverage HiFi sequencing data. Hortic. Res. 11, uhae023. doi: 10.1093/hr/uhae023 38469379 PMC10925850

[B5] Boonkerd T. Pollawatn R. (2004). A revised taxonomic account of the fern genus *Woodwardia* (Blechnaceae) in Thailand. Thai For. Bull. (Bot.) 32, 1–5. doi: 10.13140/2.1.1756.288042773082

[B6] Butenko A. Lukeš J. Speijer D. Wideman J. G. (2024). Mitochondrial genomes revisited: Why do different lineages retain different genes? BMC Biol. 22, 15. doi: 10.1186/s12915-024-01824-1 38273274 PMC10809612

[B7] Cai Z. Wu Y. Xu L. Zhao R. Wang B. Kang T. (2016). Identification of *Dryopteridis crassirhizomatis* rhizome based on *psbA*-*trnH* barcode. China J. Chin. Mater. Med. 41, 4183–4187. doi: 10.4268/cjcmm20162216 28933086

[B8] Camacho C. Coulouris G. Avagyan V. Ma N. Papadopoulos J. Bealer K. . (2009). BLAST+: architecture and applications. BMC Bioinf. 10, 421. doi: 10.1186/1471-2105-10-42 20003500 PMC2803857

[B9] Cárdenas G. G. Lehtonen S. (2023). The third opinion on fern phylogenetics with novel insights into their mitogenome evolution. Phytotaxa 625, 227–247. doi: 10.11646/phytotaxa.625.3.1

[B10] Castresana J. (2000). Selection of conserved blocks from multiple alignments for their use in phylogenetic analysis. Mol. Biol. Evol. 17, 540–552. doi: 10.1093/oxfordjournals.molbev.a026334 10742046

[B11] Cauz-Santos L. A. (2025). Beyond conservation: The landscape of chloroplast genome rearrangements in angiosperms. New Phytol. 247, 2571–2580. doi: 10.1111/nph.70364 40613318 PMC12371153

[B12] Chen C. Wu Y. Li J. Wang X. Zeng Z. Xu J. . (2023a). TBtools-II: A “one for all, all for one” bioinformatics platform for biological big-data mining. Mol. Plant 16, 1733–1742. doi: 10.1016/j.molp.2023.09.010 37740491

[B13] Chen S. Yin X. Han J. Sun W. Yao H. Song J. . (2023b). DNA barcoding in herbal medicine: Retrospective and prospective. J. Pharm. Anal. 13, 431–441. doi: 10.1016/j.jpha.2023.03.008 37305789 PMC10257146

[B14] Chiu P. S. (1974). On the genus *Woodwardia* Sm. from the mainland of Asia. J. Syst. Evol. 12, 237–248.

[B15] Doyle J. J. Doyle J. L. (1987). A rapid DNA isolation procedure for small quantities of fresh leaf tissue. Phytochem. Bull. 19(1), 11–15.

[B16] Editorial Committee of Zhonghua Bencao State Administration of Traditional Chinese Medicine (1999). Zhonghua Bencao (Shanghai: Shanghai Scientific and Technical Press).

[B17] Emms D. M. Kelly S. (2019). OrthoFinder: Phylogenetic orthology inference for comparative genomics. Genome Biol. 20, 238. doi: 10.1186/s13059-019-1832-y 31727128 PMC6857279

[B18] Fang M. Ma J. Zhou M. Huang J. Zhu W. Han L. . (2026). Comparative analysis of complete mitogenomes of two ornamental plants *Chimonanthus salicifolius* and *Chimonanthus praecox* in the family Calycanthaceae. BMC Plant Biol. 26, 696. doi: 10.1186/s12870-026-08260-z 41691163 PMC13085349

[B19] Flora of China Editorial Committee (2013). Flora of China, Vols. 2–3: Lycopodiaceae Through Polypodiaceae (Beijing: Science Press & St. Louis, MO: Missouri Botanical Garden Press).

[B20] Greiner S. Lehwark P. Bock R. (2019). OrganellarGenomeDRAW (OGDRAW) version 1.3.1: Expanded toolkit for the graphical visualization of organellar genomes. Nucleic Acids Res. 47, W59–W64. doi: 10.1093/nar/gkz238 30949694 PMC6602502

[B21] Guo W. Zhu A. Fan W. Mower J. P. (2017). Complete mitochondrial genomes from the ferns *Ophioglossum californicum* and *Psilotum nudum* are highly repetitive with the largest organellar introns. New Phytol. 213, 391–403. doi: 10.1111/nph.14135 27539928

[B22] Hao W. Liu G. Wang W. Shen W. Zhao Y. Sun J. . (2021). RNA editing and its roles in plant organelles. Front. Genet. 12, 757109. doi: 10.3389/fgene.2021.757109 34659369 PMC8511385

[B23] Hershberg R. Petrov D. A. (2008). Selection on codon bias. Annu. Rev. Genet. 42, 287–297. doi: 10.1146/annurev.genet.42.110807.091442 18983258

[B24] Huang Y. Men W. Xing Y. Hou W. Huang Y. Yang Y. . (2024). The complete chloroplast genome of *Blechnopsis orientalis* (Linnaeus) C. Presl 1753 (Blechnaceae). Mitochondrial DNA Part B 9, 986–990. doi: 10.1080/23802359.2024.2385618 39108545 PMC11302461

[B25] Huang Y. Xing Y. Men W. Xue H. Hou W. Huang Y. . (2025). The first complete mitochondrial genome assembly and comparative analysis of the fern Blechnaceae family: *Blechnopsis orientalis*. Front. Plant Sci. 16, 1534171. doi: 10.3389/fpls.2025.1534171 40182550 PMC11965612

[B26] Katoh K. Standley D. M. (2013). MAFFT multiple sequence alignment software version 7: Improvements in performance and usability. Mol. Biol. Evol. 30, 772–780. doi: 10.1093/molbev/mst010 23329690 PMC3603318

[B27] Knoop V. (2023). C-to-U and U-to-C: RNA editing in plant organelles and beyond. J. Exp. Bot. 74, 2273–2294. doi: 10.1093/jxb/erac488 36527364

[B28] Kumar A. Giri P. Uniyal P. L. (2022b). “ Genome evolution in ferns: Molecular phylogenomics – a review,” in Ferns: Biotechnology, Propagation, Medicinal Uses and Environmental Regulation. Eds. Marimuthu J. Fernández H. Kumar A. Thangaiah S. (Singapore: Springer Nature), 13–37.

[B29] Kumar S. Suleski M. Craig J. M. Kasprowicz A. E. Sanderford M. Li M. . (2022a). TimeTree 5: An expanded resource for species divergence times. Mol. Biol. Evol. 39, msac174. doi: 10.1093/molbev/msac174 35932227 PMC9400175

[B30] Li H. (2018). Minimap2: Pairwise alignment for nucleotide sequences. Bioinformatics 34(18), 3094–3100. doi: 10.1093/bioinformatics/bty191 29750242 PMC6137996

[B31] Li D. Li H. Yang L. Luo X. Chang H. Xu X. . (2025). Comparative analysis of chloroplast genomes within *Saxifraga* sect. Ciliatae: insights into their adaptation to the alpine environment. BMC Plant Biol. 25, 884. doi: 10.1186/s12870-025-06706-4 40615780 PMC12228397

[B32] Lukeš J. Kaur B. Speijer D. (2021). RNA editing in mitochondria and plastids: Weird and widespread. Trends Genet. 37, 99–102. doi: 10.1016/j.tig.2020.10.004 33203574

[B33] Ma R. Pan H. Shen T. Li P. Chen Y. Li Z. . (2017). Interaction of flavonoids from *Woodwardia unigemmata* with bovine serum albumin (BSA): Application of spectroscopic techniques and molecular modeling methods. Molecules 22, 1317. doi: 10.3390/molecules22081317 28792461 PMC6152408

[B34] Mower J. P. Sloan D. B. Alverson A. J. (2012). “ Plant mitochondrial genome diversity: The genomics revolution,” in Plant Genome Diversity, Volume 1: Plant Genomes, Their Residents, and Their Evolutionary Dynamics. Eds. Wendel J. F. Greilhuber J. Dolezel J. Leitch I. J. ( Vienna: Springer), 123–144.

[B35] Nguyen L. T. Schmidt H. A. von Haeseler A. Minh B. Q. (2015). IQ-TREE: A fast and effective stochastic algorithm for estimating maximum-likelihood phylogenies. Mol. Biol. Evol. 32, 268–274. doi: 10.1093/molbev/msu300 25371430 PMC4271533

[B36] Palmer J. D. (1985). Comparative organization of chloroplast genomes. Annu. Rev. Genet. 19, 325–354. doi: 10.1146/annurev.ge.19.120185.001545 3936406

[B37] Palmer J. D. Herbon L. A. (1988). Plant mitochondrial DNA evolves rapidly in structure, but slowly in sequence. J. Mol. Evol. 28, 87–97. doi: 10.1007/BF02143500 3148746

[B38] Park H. S. Jayakodi M. Lee S. H. Jeon J. H. Lee H. O. Park J. Y. . (2020). Mitochondrial plastid DNA can cause DNA barcoding paradox in plants. Sci. Rep. 10, 6112. doi: 10.1038/s41598-020-63233-y 32273595 PMC7145815

[B39] Patel R. K. Jain M. (2012). NGS QC Toolkit: A toolkit for quality control of next generation sequencing data. PloS One 7, e30619. doi: 10.1371/journal.pone.0030619 22312429 PMC3270013

[B40] Peng Y. Wang Z. Li M. Wang T. Su Y. (2024). Characterization and analysis of multi-organ full-length transcriptomes in *Sphaeropteris brunoniana* and *Alsophila latebrosa* highlight secondary metabolism and chloroplast RNA editing pattern of tree ferns. BMC Plant Biol. 24, 73. doi: 10.1186/s12870-024-04746-w 38273309 PMC10811885

[B41] Qu X. J. Moore M. J. Li D. Z. Yi T. S. (2019). PGA: A software package for rapid, accurate, and flexible batch annotation of plastomes. Plant Methods 15, 50. doi: 10.1186/s13007-019-0435-7 31139240 PMC6528300

[B42] Rambaut A. Drummond A. J. Xie D. Baele G. Suchard M. A. (2018). Posterior summarization in Bayesian phylogenetics using Tracer 1.7. Syst. Biol. 67, 901–904. doi: 10.1093/sysbio/syy032 29718447 PMC6101584

[B43] Ramekar R. V. Choi I. Y. Cheong E. J. Kwak M. Park K. C. (2020). The complete chloroplast genome of *Woodwardia japonica*. Mitochondrial DNA Part B 5, 102–103. doi: 10.1080/23802359.2019.1698366 33366442 PMC7748671

[B44] Singh S. Naik J. Pandey A. (2022). Plant Genomics for Sustainable Agriculture (Singapore: Springer Singapore).

[B45] Sun N. Han F. Wang S. Shen F. Liu W. Fan W. . (2024a). Comprehensive analysis of the *Lycopodium japonicum* mitogenome reveals abundant tRNA genes and cis-spliced introns in Lycopodiaceae species. Front. Plant Sci. 15, 1446015. doi: 10.3389/fpls.2024.1446015 39228832 PMC11368720

[B46] Sun W. Wei Z. Gu Y. Wang T. Liu B. Yan Y. (2024b). Chloroplast genome structure analysis of *Equisetum* unveils phylogenetic relationships to ferns and mutational hotspot region. Front. Plant Sci. 15, 1328080. doi: 10.3389/fpls.2024.1328080 38665369 PMC11044155

[B47] Takuli P. Khulbe K. Kumar P. Parki A. Syed A. Elgorban A. M. (2020). Phytochemical profiling, antioxidant and antibacterial efficacy of a native Himalayan fern *Woodwardia unigemmata* (Makino) Nakai. Saudi J. Biol. Sci. 27, 1961–1967. doi: 10.1016/j.sjbs.2020.06.006 32714019 PMC7376181

[B48] Talavera G. Castresana J. (2007). Improvement of phylogenies after removing divergent and ambiguously aligned blocks from protein sequence alignments. Syst. Biol. 56, 564–577. doi: 10.1080/10635150701472164 17654362

[B49] Testo W. Sundue M. (2016). A 4000-species dataset provides new insight into the evolution of ferns. Mol. Phylogenet. Evol. 105, 200–211. doi: 10.1016/j.ympev.2016.09.003 27621129

[B50] The Pteridophyte Phylogeny Group (2016). A community-derived classification for extant lycophytes and ferns. J. Syst. Evol. 54, 563–603. doi: 10.1111/jse.1222940046247

[B51] Thiel T. Michalek W. Varshney R. Graner A. (2003). Exploiting EST databases for the development and characterization of gene-derived SSR markers in barley (*Hordeum vulgare* L.). Theor. Appl. Genet. 106, 411–422. doi: 10.1007/s00122-002-1031-0 12589540

[B52] Wang X. Cao J. Tian L. Liu B. Fan Y. Wang Q. (2023). Elucidating flavonoid and antioxidant activity in edible and medicinal herbs *Woodwardia japonica* (L.f.) Sm. based on HPLC-ESI-TOF-MS and artificial neural network model: Response to climatic factors. Molecules 28, 1985. doi: 10.3390/molecules28041985 36838973 PMC9964229

[B53] Wang J. Kan S. Liao X. Zhou J. Tembrock L. R. Daniell H. . (2024). Plant organellar genomes: Much done, much more to do. Trends Plant Sci. 29, 754–769. doi: 10.1016/j.tplants.2023.12.014 38220520

[B54] Wang D. Zhang Y. Zhang Z. Zhu J. Yu J. (2010). KaKs_Calculator 2.0: A toolkit incorporating gamma-series methods and sliding window strategies. Genomics Proteomics Bioinf. 8, 77–80. doi: 10.1016/S1672-0229(10)60008-3 20451164 PMC5054116

[B55] Wei R. Yan Y. H. Harris A. J. Kang J. Shen H. Xiang Q. P. . (2017). Plastid phylogenomics resolve deep relationships among Eupolypod II ferns with rapid radiation and rate heterogeneity. Genome Biol. Evol. 9, 1646–1657. doi: 10.1093/gbe/evx107 28854625 PMC5534337

[B56] Wick R. R. Schultz M. B. Zobel J. Holt K. E. (2015). Bandage: Interactive visualization of de novo genome assemblies. Bioinformatics 31, 3350–3352. doi: 10.1093/bioinformatics/btv383 26099265 PMC4595904

[B57] Wolf P. Roper J. (2008). “ Structure and evolution of fern plastid genomes,” in Cambridge (Cambridge: Cambridge University Press).

[B58] Xie D. F. Yu H. X. Price M. Xie C. Deng Y. Q. Chen J. P. . (2019). Phylogeny of Chinese *Allium* species in section Daghestanica and adaptive evolution of *Allium* (Amaryllidaceae, Allioideae) species revealed by the chloroplast complete genome. Front. Plant Sci. 10, 460. doi: 10.3389/fpls.2019.00460 31114591 PMC6503222

[B59] Yang Z. (2007). PAML 4: Phylogenetic analysis by maximum likelihood. Mol. Biol. Evol. 24, 1586–1591. doi: 10.1093/molbev/msm088 17483113

[B60] Zhang X. F. Landis J. B. Wang H. X. Zhu Z. X. Wang H. F. (2021). Comparative analysis of chloroplast genome structure and molecular dating in Myrtales. BMC Plant Biol. 21, 219. doi: 10.1186/s12870-021-02985-9 33992095 PMC8122561

[B61] Zhang Z. Xiao J. Wu J. Zhang H. Liu G. Wang X. . (2012). ParaAT: A parallel tool for constructing multiple protein-coding DNA alignments. Biochem. Biophys. Res. Commun. 419, 779–781. doi: 10.1016/j.bbrc.2012.02.101 22390928

