# Supplementary material for: Comparative organelle genome analysis of three medicinal species of the genus Woodwardia (Blechnaceae): new insights into the phylogeny and molecular identification
Source: Front Plant Sci. 2026 Oct 7;17:1905066. doi: 10.3389/fpls.2026.1905066 (PMC13683679; doi:10.3389/fpls.2026.1905066)
Supplement: Supplementary file 1 [file DataSheet1.docx]

**Supplementary materials**

**SUPPLEMENTARY FIGURE S1** Traditional Chinese medicine decoction pieces of three *Woodwardia* species. (A) *W. japonica*; (B) *W. unigemmata*; (C) *W. prolifera.*

**SUPPLEMENTARY FIGURE S2**

Long-read sequencing coverage of the assembled mitochondrial genome of *W. japonica*.

**SUPPLEMENTARY FIGURE S3**

Long-read sequencing coverage of the assembled mitochondrial genome of *W. japonica*. (A) Enlarged whole-genome view; (B) Enlarged local view.

**SUPPLEMENTARY FIGURE S4**

Long-read sequencing coverage of the assembled mitochondrial genome of *W. unigemmata*.

**SUPPLEMENTARY FIGURE S5**

Long-read sequencing coverage of the assembled mitochondrial genome of *W. unigemmata*. (A) Enlarged whole-genome view; (B) Enlarged local view.

**SUPPLEMENTARY FIGURE S6**

Long-read sequencing coverage of the assembled mitochondrial genome of *W. prolifera*.

**SUPPLEMENTARY FIGURE S7**

Long-read sequencing coverage of the assembled mitochondrial genome of *W. prolifera*. (A) Enlarged whole-genome view; (B) Enlarged local view.

**Supplementary Methods:** Figures S2–S7.

Third-generation long-read sequencing reads were aligned to the mitochondrial genome sequences assembled in this study using minimap2, and successfully mapped reads were filtered and retained using samtools. Subsequently, the aforementioned alignment results were visualized and analyzed using MUMmer to generate complete coverage maps of the third-generation reads across the genomes.

**SUPPLEMENTARY FIGURE S8**

Inferred circular mitochondrial genome of of three *Woodwardia* species. (A) *W. japonica*; (B) *W. unigemmata*; (C) *W. prolifera.*

**SUPPLEMENTARY FIGURE S9**

Coverage depth of the mitochondrial genomes of three *Woodwardia* species and the chloroplast genome of *W. prolifera*. (A–C) Third-generation sequencing coverage depth of the mitochondrial genomes of *W. japonica*, *W. unigemmata*, and *W. prolifera*, respectively; (D) Second-generation sequencing coverage depth of the *W. prolifera* chloroplast genome.

**Supplementary Methods:** Figure S9

Third-generation long-read sequencing reads were aligned to the mitochondrial genome sequences assembled in this study using minimap2. After sorting with samtools, sequencing depth was calculated, and coverage plots were generated using a Python script. The horizontal coordinate is the mitochondrial genome length and the vertical coordinate is the coverage depth.

The illumina short sequences were compared to the chloroplast genome sequences using BWA software and finally the coverage was calculated using samtools depth. The horizontal coordinate is the chloroplast genome length and the vertical coordinate is the coverage depth.

**Table**

**SUPPLEMENTARY Table S1** Comparison of mitochondrial and chloroplast genome features among three *Woodwardia* species.

| **Genome feature** | **mitochondrial genomes** | | | **chloroplast genomes** | | |
| --- | --- | --- | --- | --- | --- | --- |
|  | ***W. japonica*** | ***W. unigemmata*** | ***W. prolifera*** | ***W. japonica*** | ***W. unigemmata*** | ***W. prolifera*** |
| Genome size (bp) | 485541 | 779752 | 564263 | 153224 | 153717 | 155328 |
| GC% | 48.24 | 48.05 | 48.4 | 43.18 | 43.21 | 42.77 |
| Genes (total/different) | 151/67 | 306/68 | 226/66 | 130/119 | 131/118 | 132/117 |
| CDS (total/different) | 47/33 | 59/33 | 45/32 | 88/85 | 88/84 | 89/84 |
| tRNA (total/different) | 86/31 | 180/32 | 140/31 | 35/30 | 35/30 | 35/29 |
| rRNA (total/different) | 18/3 | 67/3 | 41/3 | 7/4 | 8/4 | 8/4 |
| Genes with introns | 18 | 16 | 15 | 22 | 22 | 23 |
| GeneBank accession | PV999925 | PV999926 | PV999927 | NC050356 | NC028543 | PQ300107 |

Note：Total refers to the total number of genes. Different refers to the number of non-redundant genes.

**SUPPLEMENTARY Table S2** List of annotated genes in the mitochondrial and chloroplast genome of three *Woodwardia* species.

| **Group of genes** | **mitochondrial genomes** | | | **chloroplast genomes** | | |
| --- | --- | --- | --- | --- | --- | --- |
|  | ***W．japonica*** | ***W．unigemmata*** | ***W． prolifera*** | ***W． japonica*** | ***W． unigemmata*** | ***W． prolifera*** |
| ATP synthase | *atp1*(2) atp4(2) atp6 atp8 atp9* | *atp1* atp1* atp4(2) atp6* atp8 atp9* | *atp1 atp4(5) atp6 atp8 atp9* | *atpA atpB atpE atpF* atpH atpI* | *atpA atpB atpE atpF* atpH atpI* | *atpA atpB atpE atpF* atpH atpI* |
| Ubichinol cytochrome c reductase | *cob* | *cob* | *cob(2)* |  |  |  |
| Cytochrome c oxidase | *cox1* cox2* cox3(3)* | *cox1* cox2* cox3* | *cox1 cox2 cox3(4)* |  |  |  |
| Maturases | *matR*(2)* | *matR*(2)* | *matR* | *matK* | *matK* | *matK* |
| Transport membrance protein | *mttB* | *mttB* | *mttB* |  |  |  |
| NADH dehydrogenase | *nad1*** nad2****(2) nad3 nad4*** nad4L(3) nad5*** nad6 nad7*** nad9* | *nad1*** nad2**** nad3 nad4*** nad4L(12) nad5*** nad6(3) nad7*** nad9(10)* | *nad1 nad2 nad3(2) nad4(2) nad4L(4) nad5 nad6 nad7 nad9* | *ndhA* ndhB* ndhC ndhD ndhE ndhF ndhG ndhH ndhI ndhJ ndhK* | *ndhA* ndhB* ndhC ndhD ndhE ndhF ndhG ndhH ndhI ndhJ ndhK* | *ndhA* ndhB*^1^(2) ndhC ndhD ndhE ndhF ndhG ndhH ndhI ndhJ ndhK* |
| Ribosomal proteins (LSU) | *rpl2*(2) rpl5 rpl16* | *rpl2* rpl5(3) rpl16* | *rpl2 rpl5 rpl16* | *rpl14 rpl16* rpl2* rpl20 rpl21 rpl22 rpl23 rpl32 rpl33 rpl36* | *rpl14 rpl16* rpl2* rpl20 rpl21 rpl22 rpl23 rpl32 rpl33 rpl36* | *rpl14 rpl16* rpl2* rpl20 rpl21 rpl22 rpl23 rpl32 rpl33 rpl36* |
| Ribosomal proteins (SSU) | *rps1 rps3** rps3** rps4 rps7 rps10(4) rps12 rps13(2) rps14* rps19(2)* | *rps1 rps3** rps4 rps7 rps10 rps12 rps13 rps14* rps19* | *rps1 rps3 rps4 rps7 rps10 rps12 rps13 rps14 rps19* | *rps11 rps12**^1^(2) rps14 rps15 rps16* rps18 rps19 rps2 rps3 rps4 rps7 rps7 rps8* | *rps11 rps12**^1^(2) rps14 rps15 rps16* rps18 rps19 rps2 rps3 rps4 rps7(2) rps8* | *rps11 rps12**^1^(2) rps14 rps15 rps16* rps18 rps19 rps2 rps3 rps4 rps7(2) rps8* |
| Subunits of photosystem Ⅰ |  |  |  | *psaA psaB psaC psaI psaJ* | *psaA psaB psaC psaI psaJ* | *psaA psaB psaC psaI psaJ* |
| Subunits of photosystem ⅠⅠ |  |  |  | *psbA(2) psbB psbC psbD psbE psbF psbH psbI psbJ psbK psbL psbM psbN psbT psbZ* | *psbA(2) psbB psbC psbD psbE psbF psbH psbI psbJ psbK psbL psbM psbN psbT psbZ* | *psbA(2) psbB psbC psbD psbE psbF psbH psbI psbJ psbK psbL psbM psbN psbT psbZ* |
| Subunits of cytochrome b/f complex |  |  |  | *petA petB* petD* petG petL petN* | *petA petB* petD* petG petL petN* | *petA petB* petD* petG petL petN* |
| Subunits of Large subunit of rubisco |  |  |  | *rbcL* | *rbcL* | *rbcL* |
| DNA dependent RNA polymerase |  |  |  | *rpoA rpoB rpoC1* rpoC2* | *rpoA rpoB rpoC1* rpoC2* | *rpoA rpoB rpoC1* rpoC2* |
| c-type cytochrom synthesis gene |  |  |  | *ccsA* | *ccsA* | *ccsA* |
| Envelope membrane protein |  |  |  | *cemA* | *cemA* | *cemA* |
| Protease |  |  |  | *clpP*** | *clpP*** | *clpP*** |
| Subunit of Acetyl-CoA-carboxylase |  |  |  | *accD* | *accD* | *accD* |
| Translation initiation factor IF-1 |  |  |  | *infA* | *infA* | *infA* |
| Protochlorophyllide reductase |  |  |  | *chlB chlL chlN* | *chlB chlL chlN* | *chlB chlL chlN* |
| Genes of unknown functions Open Reading |  |  |  | *ycf1 ycf12 ycf2(2) ycf3** ycf4* | *ycf1 ycf12 ycf2(2) ycf3** ycf4* | *ycf1 ycf12 ycf2(2) ycf3** ycf4* |
| Ribosomal RNAs | *rrn5(10) rrn18(7) rrn26** | *rrn5(24) rrn18(24) rrn26(19)* | *rrn5(12) rrn18(17) rrn26(12)* | *rrn16(2) rrn23(2) rrn4.5 rrn5(2)* | *rrn16(2) rrn23(2) rrn4.5(2) rrn5(2)* | *rrn16(2) rrn23(2) rrn4.5(2) rrn5(2)* |
| Transfer RNAs | *trnA-TGC(2) trnC-GCA(2) trnC-GCA(2) trnD-GTC(2) trnE-TTC(6) trnF-GAA(3) trnG-GCC(3) trnG-TCC(3) trnH-GTG(3) trnH-GTG trnK-TTT(2) trnL-TAA(3) trnL-TAG(2) trnM-CAT(2) trnM-CAT(5) trnM-CAT(2) trnN-GTT(2) trnP-TGG(9) trnQ-TTG(2) trnR-ACG(6) trnR-TCG(3) trnR-TCG(3) trnR-TCT(2) trnR-TCT(2) trnS-GCT trnS-GGA trnS-TGA(2) trnV-TAC(5) trnW-CCA(3) trnY-GTA trnY-GTA* | *trnA-AGC* trnA-TGC(2) trnC-GCA(2) trnD-GTC(3) trnE-TTC(11) trnF-GAA(2) trnG-GCC(12) trnG-TCC(12) trnH-GTG(12) trnK-TTT(2) trnL-TAA(12) trnL-TAG(2) trnM-CAT(2) trnM-CAT(13) trnM-CAT(2) trnN-GTT(6) trnN-GTT(13) trnP-TGG(11) trnQ-CTG(2) trnR-ACG(7) trnR-TCG(12) trnR-TCG(12) trnR-TCG trnR-TCT trnS-GCT trnS-GGA(2) trnS-TGA(2) trnV-TAC(2) trnW-CCA(12) trnY-GTA(2) trnY-GTA(3) trnY-GTA* | *trnA-TGC(5) trnA-TGC trnC-GCA(2) trnC-GCA(2) trnD-GTC(2) trnE-TTC(13) trnF-GAA(5) trnG-GCC(4) trnG-TCC(4) trnH-GTG(4) trnK-CTT(4) trnL-TAA(4) trnL-TAG(4) trnM-CAT(6) trnM-CAT(4) trnM-CAT(4) trnN-GTT(10) trnN-GTT(2) trnP-TGG(13) trnQ-CTG(6) trnR-ACG(15) trnR-CCT trnR-TCG(4) trnR-TCG(4) trnS-GCT trnS-GGA trnS-GGA trnS-TGA(4) trnV-TAC(4) trnW-CCA(4) trnY-GTA(2)* | *trnA-UGC*(2) trnC-GCA trnD-GUC trnE-UUC trnF-GAA trnG-UCC* trnG-UCC trnH-GUG(2) trnI-CAU trnI-GAU*(2) trnL-UAA* trnL-UAG trnM-CAU trnN-GUU(2) trnP-GGG trnP-UGG trnQ-UUG trnR-ACG trnR-ACG trnR-UCG trnR-UCU trnS-GCU trnS-GGA trnS-UGA trnT-GGU trnT-UGU*(2) trnV-UAC* trnW-CCA trnY-GUA trnfM-CAU* | *trnA-UGC*(2) trnC-GCA trnD-GUC trnE-UUC trnF-GAA trnG-GCC trnG-UCC* trnH-GUG(2) trnI-CAU trnI-GAU*(2) trnL-UAA* trnL-UAG trnM-CAU trnN-GUU(2) trnP-GGG trnP-UGG trnQ-UUG trnR-ACG trnR-ACG trnR-UCG trnR-UCU trnS-GCU trnS-GGA trnS-UGA trnT-GGU trnT-UGU*(2) trnV-UAC* trnW-CCA trnY-GUA trnfM-CAU* | *trnA-UGC*(2) trnC-GCA trnD-GUC trnE-UUC trnF-GAA trnG-GCC trnG-UCC* trnH-GUG(2) trnI-CAU trnI-GAU*(2) trnL-UAA* trnL-UAG trnM-CAU trnN-GUU(2) trnP-GGG trnP-UGG trnQ-UUG trnR-ACG(2) trnR-UCG trnR-UCU trnS-GCU trnS-GGA trnS-UGA trnT-GGU trnT-UGU*(2) trnV-UAC* trnW-CCA trnY-GUA trnfM-CAU* |

Notes: *: intron number; Gene(N): Number of copies of multi-copy genes; 1trans-spliced genes.
